# Gate reflectometry of single-electron box arrays using calibrated low temperature matching networks

**DOI:** 10.1038/s41598-022-06727-1

**Published:** 2022-02-23

**Authors:** Matthew J. Filmer, Matthew Huebner, Thomas A. Zirkle, Xavier Jehl, Marc Sanquer, Jonathan D. Chisum, Alexei O. Orlov, Gregory L. Snider

**Affiliations:** 1 Department of Electrical Engineering, University of Notre Dame, Notre Dame, IN 46556 USA; 2 L3Harris, Fort Wayne, IN 46818 USA; 3 Northrop Grumman, Ogden, UT 84405 USA; 4 Univ. Grenoble Alpes, CEA, Grenoble INP, IRIG, PHELIQS, 38000 Grenoble, France

**Keywords:** Characterization and analytical techniques, Electronic devices

## Abstract

Sensitive dispersive readouts of single-electron devices (“gate reflectometry”) rely on one-port radio-frequency (RF) reflectometry to read out the state of the sensor. A standard practice in reflectometry measurements is to design an impedance transformer to match the impedance of the load to the characteristic impedance of the transmission line and thus obtain the best sensitivity and signal-to-noise ratio. This is particularly important for measuring large impedances, typical for dispersive readouts of single-electron devices because even a small mismatch will cause a strong signal degradation. When performing RF measurements, a calibration and error correction of the measurement apparatus must be performed in order to remove errors caused by unavoidable non-idealities of the measurement system. Lack of calibration makes optimizing a matching network difficult and ambiguous, and it also prevents a direct quantitative comparison between measurements taken of different devices or on different systems. We propose and demonstrate a simple straightforward method to design and optimize a pi matching network for readouts of devices with large impedance, $Z \ge 1\hbox {M}\Omega$. It is based on a single low temperature calibrated measurement of an unadjusted network composed of a single L-section followed by a simple calculation to determine a value of the “balancing” capacitor needed to achieve matching conditions for a pi network. We demonstrate that the proposed calibration/error correction technique can be directly applied at low temperature using inexpensive calibration standards. Using proper modeling of the matching networks adjusted for low temperature operation the measurement system can be easily optimized to achieve the best conditions for energy transfer and targeted bandwidth, and can be used for quantitative measurements of the device impedance. In this work we use gate reflectometry to readout the signal generated by arrays of parallel-connected Al-AlOx single-electron boxes. Such arrays can be used as a fast nanoscale voltage sensor for scanning probe applications. We perform measurements of sensitivity and bandwidth for various settings of the matching network connected to arrays and obtain strong agreement with the simulations.

## Introduction

Single-electron devices serving as non-invasive broadband (>1 MHz) sensing elements often employ the one-port radio-frequency (RF) reflectometry to read out the state of the sensor. The so-called gate reflectometry^1–5^ that utilizes existing gates of single-electron transistors (SETs) forming quantum dots (QD) as sensing probes was first used in applications as readout in spin qubit schemes with spin-to-charge conversion^1,2,6^. There it greatly simplifies the readout circuitry for the whole qubit architecture as it eliminates the need for additional SETs acting as electrometers. The parameter of interest for such “dispersive” readout is the dynamic (or tunneling) capacitance^7,8^ of the QD viewed from the gate $C_{dyn}(V_{g})$ that changes in response to a change in QD electron population. The gate-probing approach was also used to study effects of non-adiabatic dissipative processes (the “Sisyphus resistance” effect)^9^ or combinations of both, dispersive and dissipative effects^10,11^ in single-electron devices implemented in metal/metal oxide systems. Recently, gate reflectometry with predominantly dispersive response was used to read out the sensor composed of array of single-electron boxes (SEB) connected in parallel and targeting scanning probe applications^12,13^. One important constraint for dispersive readout is dictated by the tunneling rate of electrons at a degeneracy point, $\gamma _{0}=k_{B}T/e^2R$ where $R \ge {25} k$ $\Omega$ is tunnel junction resistance,and *T* is the temperature. For $f \ge \gamma$ the $C_{dyn}(V_{g})$ is greatly reduced and Sisyphus resistance effect dominates^11^. This limits the range of probing RF frequencies for dispersive readout to $\le 1$ GHz for the majority of Coulomb blockade devices operating at low temperatures (<4.2K).

**Figure 1:**
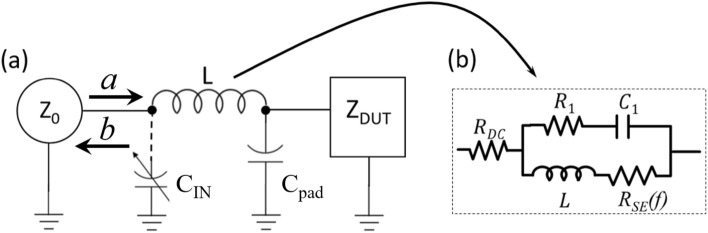
(**a**) Generic 1-port reflectometry setup. $Z_{DUT}$ is a general representation of a device connected to a MN. This impedance was treated as purely resistive in original RF-SET research^14^ where it represents device source-drain resistance $R_{{SET}} \left( {V_{g} } \right)$. For gate reflectometry work^1–5,10,11^, DUT corresponds to predominantly capacitive gate impedance. $C_{IN}$ is a tunable capacitor that enables close matching conditions and is discussed at length in this work. (**b**) Realistic model of inductors^15^ used in experiments^1–5,10,11^.

Single port RF reflectometry for nanoelectronic applications was first used to track changes in the source-drain resistance $R_{SET}$ of single-electron transistors^14^. Generally speaking, in this case the changes the impedance of the device under test (DUT), $Z_{DUT}$, are detected using a simple “L-section” matching network (MN) comprising a series off-chip inductor, *L*, and parallel bonding pad capacitance, $C_{pad}$, to ground (Fig. 1a) connected to the gate of the DUT. The use of a L-section for the MN enables measurements of DUTs impedances much larger than standard line impedance of ${50}\Omega$ ($Z_{DUT}=R_{SET}\gg Z_0$) using reflectometry. Near the resonant frequency $f_0=1/(2\pi \sqrt{LC_{pad}})$ the MN converts $Z_{DUT}=R_{SET}(V_{g}=V_{ON})\approx 100\,\hbox {k}\Omega$ into impedance of MN with the embedded DUT, $Z_{IN}$

1 $$\begin{aligned} Z_{IN}=L/(C_{pad} R_{SET})+j\omega L+1/(j\omega C_{pad}) \end{aligned}$$

Here $V_{ON}$ is a gate voltage at which Coulomb blockade is completely or partially lifted, depending on the choice of working point in $V_{g}$. By choosing an appropriate value of *L*, at resonance $Z_{IN}=L/(C_{pad} R_{SET}(V_{ON}))$ can be set to approach $Z_{0}$ and thus achieve a significant change in the reflection coefficient $\Delta \Gamma =(Z_{IN}-Z_{0})/(Z_{IN}+Z_{0}) \approx 1$^16^. The performance optimization for a sensor implies maximization of its signal-to-noise ratio (SNR), which for a drain-coupled RF SET is directly proportional to the change in the magnitude of reflection coefficient as a function of the change in $R_{SET}(V_{g})$^16^.

Similar L-section approach was used for gate-coupled devices studied in^1–5,10,11^, however, the DUT impedance in Fig. 1a is distinctly different from that of drain-coupled devices^14,16^. First, this impedance is typically many orders of magnitude larger in value at RF frequencies of interest (100–1000 MHz) ($>10$ M$\Omega$) and instead of being purely resistive, can in general be represented by a parallel combination of $G_{DUT}$ and $C_{DUT}$^11^, attributed to a combination of Sisyphus resistance^9^ and dynamic capacitance^7,8^. Clearly, to obtain $Z_{IN}$ approaching $Z_0$ would require a radical increase in the $L/C_{pad}$ ratio to balance the increase in the $Z_{DUT}$. In practice, $C_{pad} \ge {0.1}$ pF, so for large $Z_{DUT}\gg 1$ M$\Omega$ the $Z_{IN}\ll Z_{0}$ and the DUT is always overcoupled to the feedline^16^. As a result it is impossible to reach close to match conditions with the simple L-section MN. However, if the off-chip inductor is judiciously selected, an additional parallel capacitor ($C_{IN}$ in Fig. 1a) forms a $\Pi$ MN^17^ that can be designed to achieve good match for the case when $Z_{IN}\ll Z_{0}$ for $C_{IN}$=0. The simplicity of this approach with an off-chip inductor *L*, a given pad capacitor $C_{pad}$ and balancing capacitor $C_{IN}$ makes it an attractive option for the design of the MN. The most common inductors used in these applications are ceramic core surface mount inductors in the range 100–1000 nH (for example, 0805CS Coilcraft Ceramic Chip Inductors^15^) which enables the operation in the range 0.1–1 GHz. These inductors have small dimensions which allows their placement in very close proximity to the nanodevice under test. It is, however, very important to take into account the non-idealities of components used in the design of the $\Pi$ MN, with the inductor being the dominant source of non-ideality.

Recently, Ares et al.^18^ attempted to design and use a $\Pi$ MN using a 5 element circuit model (Fig. 1b). However, the parameters of the circuit model used by Ares^18^ are only known at $T=293$ K, while direct characterization of inductors reveals a very significant change in the DC resistance of the inductor with lowering temperature (by a factor $\sim 70$ for $T \le 10$ K^13^) and there are likely changes in the parameters of other parasitic elements, so that no accurate quantitative comparison is possible between the 300 K model and experimental results at $T\le 10$ K. Since the value of $C_{IN}$ in this case cannot be accurately calculated to obtain close match, Ares et al.^18^ utilized a varactor as a variable capacitor $C_{IN}$ to empirically reach a matching condition. They demonstrated that by obtaining a close match the sensitivity of the circuit to the variations in device capacitance can be improved. Their results also indicate that below 1 K the GaAs varactor has diminished range of operation due to carrier freeze out. To avoid this problem, a fixed-value capacitor can be used instead. However, this requires a good understanding of the low temperature behavior of the MN components. Indeed, even if a good match could be accurately predicted at room temperature using manufacturer’s models, the parasitics of the inductors exhibit a strong temperature dependence, thus changing the parameters of the network which in turn results in a different value of $C_{IN}$ required to achieve match. The importance of properly accounting for parasitic elements of the circuit and their temperature dependence was clearly demonstrated in recent publications^13,19^.

As we show below, proper account for parasitic parameters also has very significant impact on the accessible bandwidth (see part S3, S4, and S7 in Supplementary materials for more details). This shows the paramount importance of proper model considerations in avoiding gross errors in the design of matching network and, consequently, interpreting the results of experiments. In this context, one important issue which is often overlooked in the experimental literature is the need for a properly calibrated experimental RF setup. Reflectometry measurements can only be quantitatively correct if the measurement system is calibrated against known standards. This means, for example, that at a calibrated reference plane, both open and short standards are expected to produce $|\Gamma |=1$ and a matched load should produce $|\Gamma |=0$. Experimental setups never produce these results without the use of error correction because of a wide range of non-idealities in the signal paths (standing wave resonances in the transmission lines, deviations from exact $Z_0$, matched values in components used, frequency dependent transfer characteristics and phase shifts in the amplifiers and couplers, etc.). To be able to accurately correlate RF measurements to models, it is therefore critical to error correct the measured data through calibration, a standard practice when measuring S-parameters with a network analyzer. The Calibration and Error Correction (CEC) is a standard practice for room temperature RF measurements, and room-temperature RF instrumentation^20^ but it is not typically performed for low temperature reflectometry, and uncalibrated data are, with a few exceptions^21^, reported in the literature. Here we demonstrate that the standard Short-Open-Load CEC protocol can be directly applied at low temperature using inexpensive calibration standards and thus a) greatly simplify the design of MN and b) obtain the data that can be quantitatively compared with the DUT models. Strictly speaking, without proper calibration only qualitative analysis is possible.

**Figure 2:**
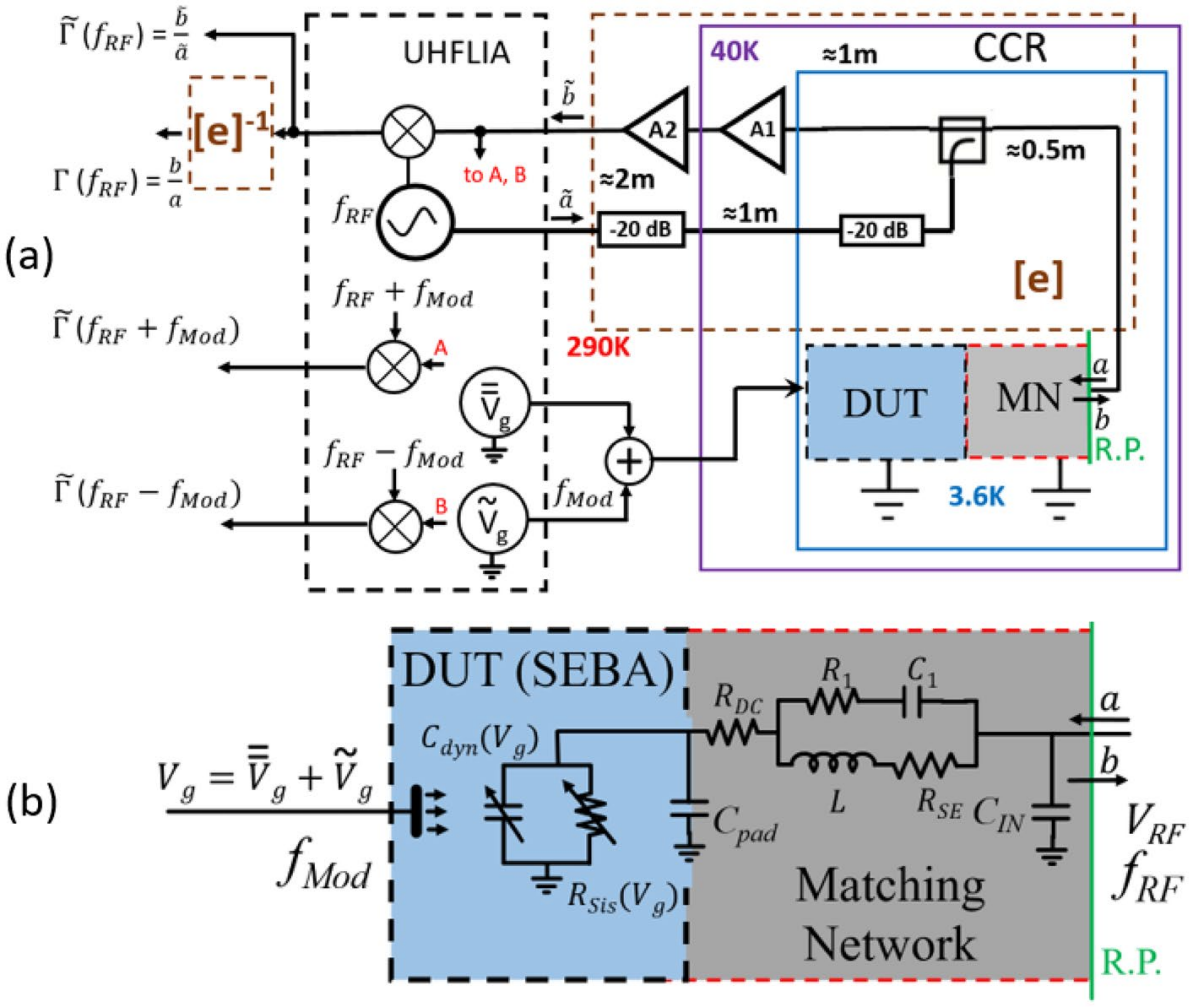
(**a**) Experimental setup. Components surrounded by a violet line are inside close cycle refrigerator (CCR), with cold amplifier A1 at 40 K. Components within blue box are at T = 3.6 K. Dashed brown box labeled “[e]^−1^” contains all the components that contribute to non-idealities (”errors”) in the reflectometry process. Dashed brown box labeled ”[e]^−1^” is an ”error-correction box” implemented in software that generates error corrected data (ECD) by using calibrated standards to calculate error correction coefficients. UHFLIA is ZI UHF lock-in amplifier which generates all signals and performs demodulation. Dashed green line delineates the reference plane (R.P.). (**b**) Zoom-in into a combination of DUT and MN showing all of the circuit components. DUT is represented by SEBA; the SEBA admittance is controlled by the gate voltage $V_{g}$. Pad capacitance, $C_{pad}$, is shown as an element shared between MN and DUT.

The goal of this work is to develop a simple method to construct high quality, resonant MNs for measuring large impedances $\ge$ 10M$\,\Omega$ typical for gate-sensing reflectometry and accurately evaluate its sensitivity and the available measurement bandwidth using a well-characterized DUT.

**Figure 3:**
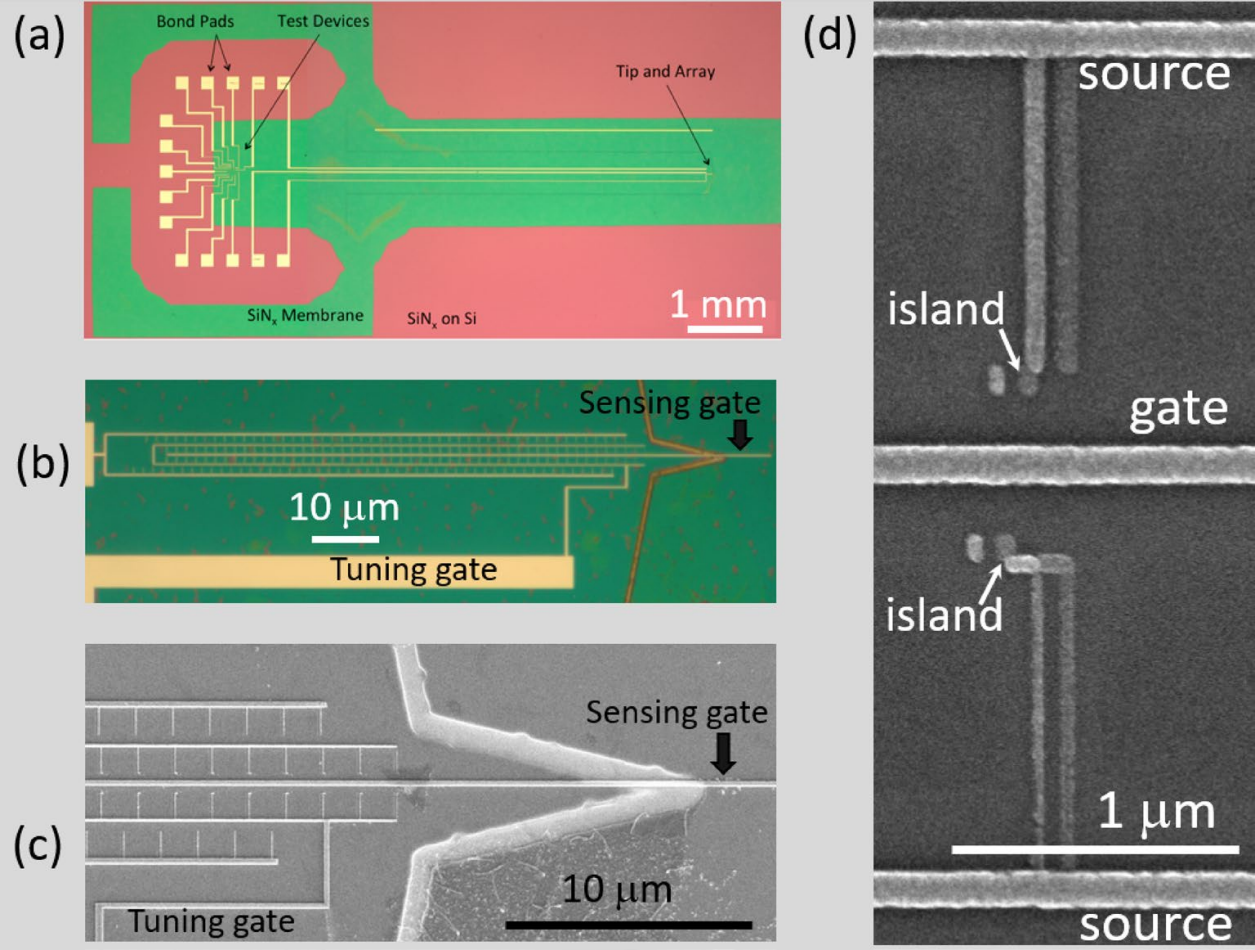
Micrographs of $Al-AlO_{X}$ SEBA fabricated using Dolan bridge technique (**a**) Optical micrograph of the SEBA on $SiN_{X}$ membrane (green) fabricated on top of oxidized Si wafer. (**b**) Zoom-in view showing SEBA with 2 gates (sensing and tuning). The trench defining the tip is etched into Si membrane using reactive ion etching (**c**) Electron micrograph of SEBA (30 SEBs are visible out of 200 total) on $SiN_{X}$ substrate. (**d**) Electon micrograph of two SEBs, the sensing gate is in the middle. All of the devices studied in this work are fabricated on fused silica substrate.

In this work we utilize arrays of Al-AlO$_{x}$ single-electron boxes^22^ (SEBA) connected in parallel^12,13^ shown in Fig.3a–c fabricated on fused silica substrates. The impedance of SEB can be adequately modeled as a parallel combination of $R_{DUT}$ and $C_{DUT}$ using a model proposed in^11^. The target application of this device is a voltage sensitive high-speed scanning probe^12,13,23,24^. The use of parallel-connected single-electron devices sharing common gate results in incoherent oscillation pattern^25^ in the amplitude of Coulomb blockade oscillations that scales up as $N^{1/2}$. In case of SEBA the use of arrays with $N \ge 100$ makes the magnitude of composite response comparable to that of an RF-SET^12,13^.

Importantly, the $Al-AlO_{x}$ single-electron boxes^9,12,13^ fabricated by Dolan-bridge technique^26^ have much higher tolerance to electrostatic discharge events compared to other (e.g. fabricated by CMOS^1–3^) single-electron devices. Since there is no DC current path through SEB, there is no DC voltage across the junction. However, if a step voltage, $V_{g}$, is abruptly applied to an SEB, it will be divided between $C_{g}$ and $C_{J}$ until $C_{J}$ discharges through $R_{J}$ so that the voltage across the junction can instantaneously reach $V_{J}\approx C_{J}/(C_{J}+C_{g}) \times V_{g}$. Here $C_{g}$ is gate capacitance and $C_{J}$ and $R_{J}$ are junction capacitance and resistance. The gate capacitor of SEB $C_{g}$ is formed by the metal line separated (horizontal line in the middle of Fig. 3d) from SET island by a thick (t $\ge$ 100 nm) dielectric with a high breakdown voltage while the junction ($C_{J}$, $R_{J}$) is formed by the source wire overlapping the metal island with tunnel-transparent dielectric (t $\approx$ 1 nm) in between. For the devices studied in this work $C_{J}/C_{g} \ge 10$, so that if a voltage is abruptly applied across SEB, the voltage across the junction is attenuated by a factor of $\ge 10$. The experimental evidence confirms that arrays of single-electron boxes studied in this work provide a robust DUT for our study which can be subjected to multiple cooling/heating cycles and can withstand soldering/desoldering of electronic components attached to ”live” devices.

## Experimental method

### Experimental setup

A simplified schematic diagram of the experimental setup used in this work is presented in Fig. 2a. Low temperature data are obtained in a closed cycle refrigerator with a base temperature of 3.6 K (by Advanced Research Systems). Signal generation and data acquisition are performed using a digital Zurich Instruments UHF Lock-In Amplifier (UHF LIA) capable of full homodyne and modulation detection of signals up to 600 MHz^27^. The UHF LIA produces the forward wave of the reflectometer ${\widetilde{a}}$ at $f_{RF}$ which goes through a -20dB attenuator at room temperature, another -20 dB attenuator by CRYSTEK and a directional coupler (ZFDC-20-50+) thermally anchored at the second stage at 3.6K to produce the incident wave *a* at the reference plane (RP) of the MN and DUT. The reflected wave *b* returns through the system to the UHF LIA (${\widetilde{b}}$). To combat the accumulated losses and improve the SNR we use low noise amplifiers by Minicircuits: the first stage, ZX60-P33ULN+, (A1 in Fig. 2a) located at the 1st stage of the cryocooler at T $\approx$ 40 K, and two stages, ZX60-P103LN+, at room temperature (A2 in Fig. 2a), for a total gain $\approx {70}$  dB at 500 MHz. Special care is taken to avoid the variation of gain over time in both cold and warm amplifiers, for which regulated power supplies with stable output voltage (+ 5 V for warm amplifiers and + 2.7 V for cold amplifiers) are used throughout the experiment.

The system calibration and error correction is performed as described below in “Calibration and error correction”. As a result of CEC, the errors between reference plane and input of the UHF LIA are eliminated. This enables experimental determination of MN settings that achieves a match using a technique described in details in “Optimization of matching network” employing a minimal knowledge of parasitic parameters of the inductor *L* and $C_{pad}$ used in $\Pi$ MN for a variety of high impedance DUTs, $Z_{DUT} \ge {10}\hbox {M}\Omega$.

For the experiments described in “Gate reflectometry: sensitivity and bandwidth tuning” we utilize the UHF-MOD AM/FM Modulation option in digital ZI UHF^27^ which allows simultaneous extraction of sidebands (SB) for the amplitude modulated signal applied to the gates of SEBA by performing synchronous demodulation at three frequencies: $f_{RF}-f_{Mod}$, $f_{RF}$, and $f_{RF}+ f_{Mod}$. For that purpose a small modulating signal $\tilde{V_{g}}$ superimposed with DC gate bias $\bar{\bar{V}}_{g}$ is applied to the gates of SEBA. In this work we do not apply CEC protocol for the SB signals. The ZI UHF lock-in allows implementation of error correction on SB before the demodulators, this option will be explored in our future work.

### Calibration and error correction

In this work, we follow a one port *Short*-*Open*-*Load* (SOL) calibration/error correction protocol. To do so a collection of three known terminations are prepared and measured on a printed circuit board identical to the actual board containing MN elements and DUT (i.e., the calibration board is identical by design to the one used in the experiment, fabricated in the same batch from a single drawing file) and error correction coefficients are computed for chosen temperatures (in the context of this paper, 300 K and 3.6 K. The detailed description of error correction protocol, SOL calibration kit and its use are presented in part S1 of the Supplementary materials. All circuit elements are inside the brown dashed box, labeled ”e” in Fig. 2a (i.e. cables, amplifiers, directional coupler, attenuators) introduce unavoidable errors due to their frequency dependent characteristics. By performing calibration we create an ”error-correction box”, labeled ”[e]^−1^” in Fig. 2a so that the acquired raw data can be error-corrected to yield an ”error-free” $\Gamma (f_{RF})$ signal. By using error correction we in effect move the reference plane from the input of UHF LIA to the output of the reflecting element, i.e. to the MN with the attached DUT (Fig. 2b). The green dashed line in Fig. 2a indicates the position of the reference plane after CEC; in practice this corresponds to the solder joint of the inductor *L* and balancing capacitor $C_{IN}$ (see Fig. S3 in Supplementary materials). An example of experimental data before and after error correction is presented in Fig. S4 in Supplementary materials.

### Optimization of matching network

The design of any circuit begins with a reliable model of the actual components being used. A minimal knowledge of the pad capacitance enables crude evaluation of the desired operating frequency based upon available inductor values: *f* = 1/2$\pi \sqrt{LC}$ (the actual resonance frequency can be up to 20% lower due to parasitic parallel components, Fig. 1b).

In our experiments, the value of $C_{pad}$ is on the order of 0.5 pF. Here we accumulated all capacitances to ground in parallel with the DUT (e.g. bond wire to ground) into $C_{pad}$.

**Figure 4:**
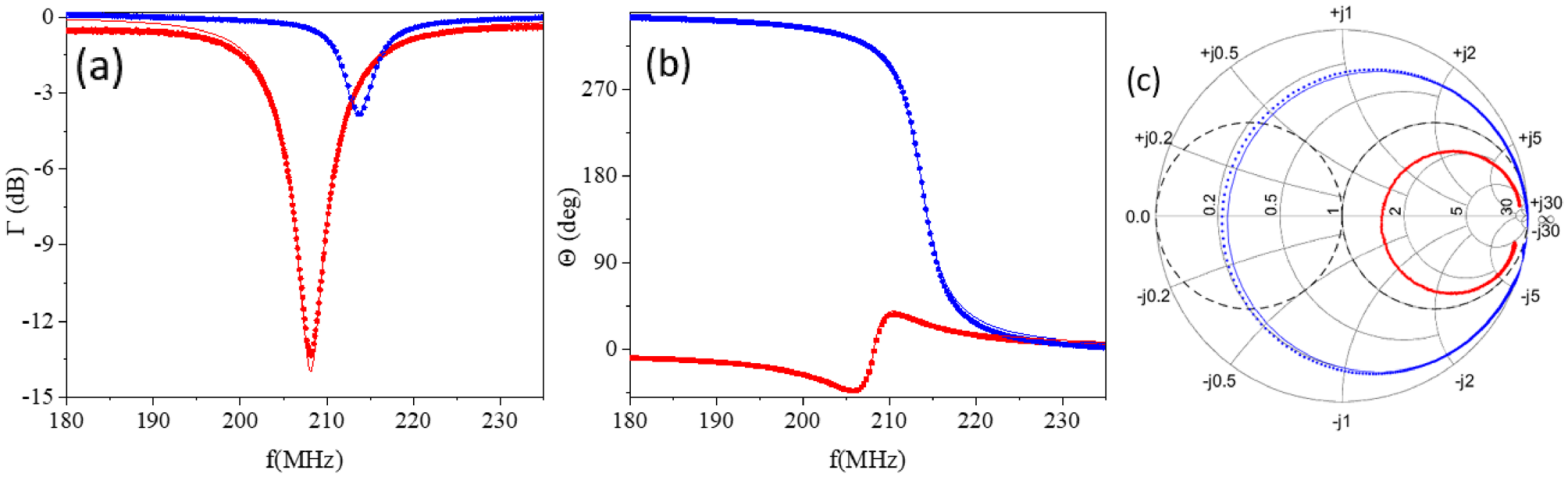
Results of reflectometry measurements at T=300K and 3.6K for MN composed of L=820 nH inductor and $C_{pad}$ with no DUT present; error-corrected data are shown: (**a**) magnitude measurements; (**b**) phase measurements; (**c**)-Smith chart. Dots—experiment, solid lines—calculations. Red: 300 K, blue: 3.6 K. Simulations are performed based on 820 nH inductor Coilcraft model^15^ adjusted for low temperature.

To construct the MN we use off the shelf inductors by Coilcraft^15^with unknown parasitics at low temperature. The selected inductor is installed on the board, thus creating a DUT + MN combination attached at a reference plane as shown Fig. 2b. The frequency response of the partially assembled MN is acquired at the intended operation temperature for which calibration is performed and error-correction is applied. To illustrate the change in the tuning of MN caused by temperature the experiment that involves only *L* and $C_{pad}$, i.e with no $Z_{DUT}$ connected, is performed. Note that the capacitance of the DUT studied in this work is at least $10^3$ smaller than $C_{pad}$ so the change in the reflected signal with DUT added would be below the resolution limit in Fig. 4. The results of the experiment for *L* = 820 nH and $C_{pad}$ are presented in Fig. 4 where magnitude and phase plotted vs frequency (Fig. 4a,b) and in the Smith chart (Fig. 4c) for two temperatures, 300 K (in red), and 3.6 K (in blue).

The data acquired at room temperature (red dots in Fig. 4) are in very good correlation with the Coilcraft model^15^ for the $L=$ 820 nH inductor and $C_{pad}=$ 458 fF, with $C_{pad}$ being the only adjustable parameter which sets the operating frequency. However, the data acquired at low temperature 3.6 K (blue dots in Fig. 4) clearly indicate that parameters of the MN change significantly with temperature. This presents a challenge when trying to design a MN using components for which good models are only available at room temperature. Fortunately, precise model parameters are not needed if the impedance can be measured directly using an error-corrected system. To better understand the change in parasitic components of the inductor caused by the reduction of temperature we performed simulations of the frequency response of MN composed of $LC_{pad}$ section and compared it with the experiment (see Supplementary materials, Sect. S5 for details). In simulations we took into account experimentally observed reduction of the series coil resistance due to reduction of resistivity and associated reduction of skin-effect resistance^13^. The results of the simulations indicate that the components associated with core loss ($R_{1},C_{1}$ in Fig. 1b) also change with temperature. These changes in the MN parameters result in the expansion of the impedance trajectory vs frequency in the Smith chart out of $r=1$ circle with lowering of the temperature. This change in the impedance trajectory is crucially important for the success of the proposed method limiting it to the designs of MNs for which $Z_{IN}<Z_{0}$, i.e when $Z_{IN}$ without balancing capacitor (i.e.for $C_{IN}=0$ ) is overcoupled to the feedline at operation temperature; otherwise different MN solutions may be applied but they are beyond the scope of this paper.

**Figure 5:**
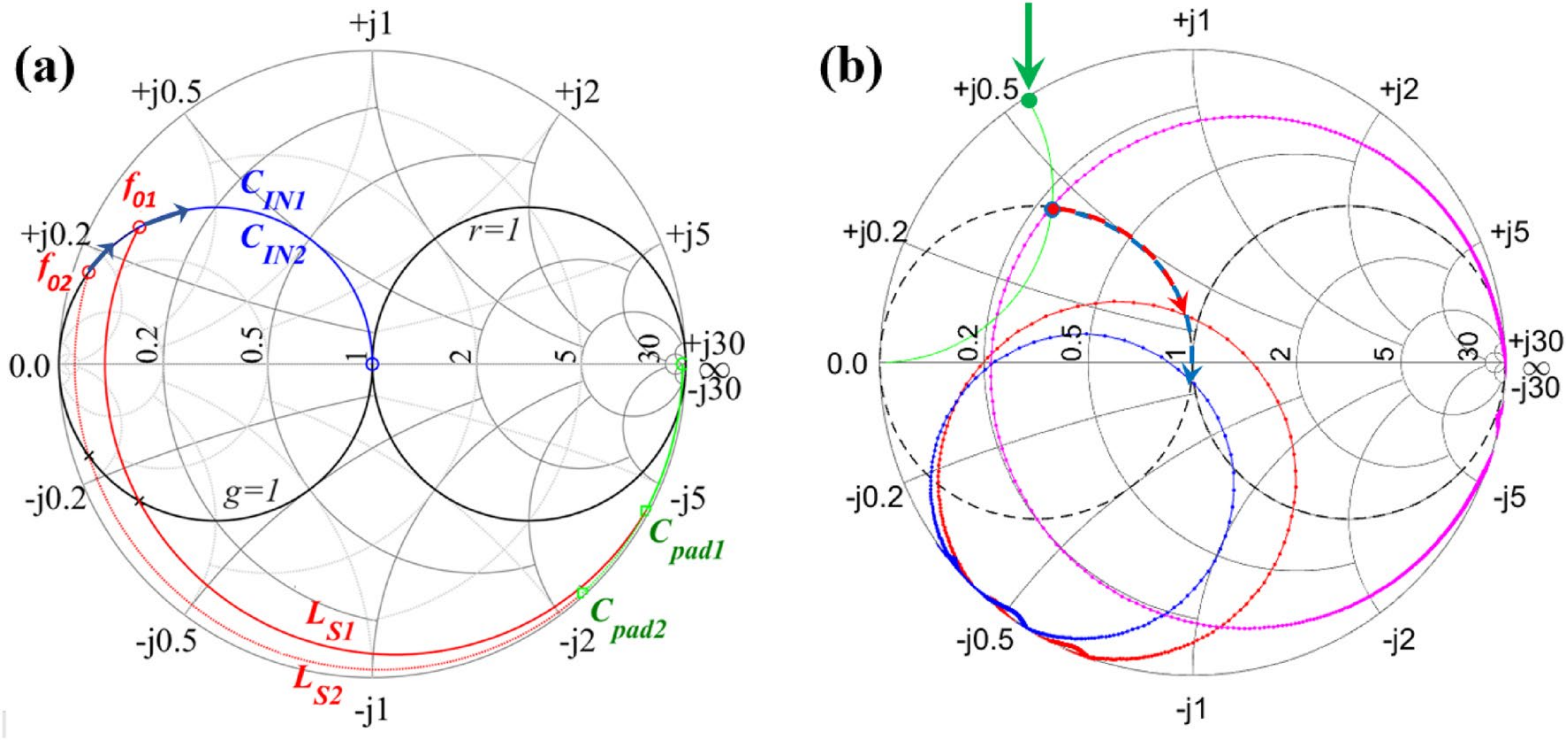
(**a**) The impedance trajectory sketch showing how to obtain the matched load. Green arcs represent the resulting effect of pad capacitance $C_{pad}$ added in parallel with DUT. Two cases are shown for two different values of pad capacitance, with a shorter green arc corresponding to a smaller $C_{pad}$. (**b**) Experimental results obtained using ECD. Magenta arc represent the result of reflectometry measurement shown in Fig. 4 for 3.6 K. Green line towards the periphery follows the constant susceptance curve and reaches the edge of the Smith chart at the point indicated by a green dot and green arrow. This value of susceptance is used in calculation of required $C_{IN}$ = 24 pF which moves the impedance to the center of the Smith chart (blue arrows). Blue arc—experimental data for $C_{IN}$ = 27 pF; red arc—$C_{IN}$ = 20 pF.

Figure 5a illustrates the procedure we utilize to determine the value of the component $C_{IN}$ in the MN. We will start our analysis with the (unrealistic) case when $C_{pad}$ = 0 and then add components to modify $Z_{IN}$. Initially, since $Z_{DUT} \gg Z_0$, the impedance trajectory vs carrier frequency is situated very close to the right border of the Smith chart, within the $r=1$ circle in Fig. 5 making it very far away from the matching point. However, parasitic pad capacitance $C_{pad}$ moves the impedance trajectory outside the $r=1$ circle, green arcs in Fig. 5a. Two green arcs are shown in Fig. 5a with a longer arc corresponding to a larger $C_{pad}$, which in practice may correspond to the same size pad but on a different substrate (for example, switching from SiO$_{2}$ to SiN$_{x}$ for $C_{pad}$ results in the increase of dielectric constant by a factor of 9.5/3.9$\,\approx {2.4}$ thus increasing the $C_{pad}$ by the same amount). Alternatively, the same analysis can be applied for the same $C_{pad}$ at two different target frequencies, with longer arc corresponding to the higher frequency. To reach matching condition an impedance transformer is required to raise the real-part of the impedance. A series inductor $L_{S1}$ moves the impedance trajectory clockwise along a constant resistance circle. The addition of the inductor needs to move the impedance trajectory inside the Smith chart so that it intersects the matched admittance circle $g=1$ at some frequency $f_{01}$ in the upper half of the Smith chart, corresponding to a capacitance in parallel with $L_{S1}C_{pad}$ section. That frequency, $f_{01}$, becomes the operating frequency of the matching network, $f_{Op}$. Depending on the application, for a different $C_{pad}$ the user may either choose a different inductor (e.g., a $L_{S2}<L_{S1}$ for a larger $C_{pad}$ in Fig. 5a) so that the operating frequency stays approximately the same $f_{01}\approx {f_{02}}$, or keep the same inductor $L_{S1}$ and allow operating frequency to change. It is important to note that in case of $Z_{DUT} \gg 1M$ this is possible only if added inductor has some parasitic dissipative component on the order of $Z_{0}$ in form of coil resistance or loss in the core, which is exactly the case for the inductors used in this work. Otherwise, the expanded impedance trajectory runs too close to the edge of the Smith chart and as we show below determination of $C_{pad}$ using the proposed method becomes problematic.

Once the crossing of the $g=1$ circle is ensured, a balancing capacitor $C_{IN}$ in parallel with $LC_{pad}$ is introduced to eliminate the remaining reactance and thus move the impedance trajectory to the center of the Smith chart at $f_{Op}$. To compute the required $C_{IN}$, the susceptance *b* of MN at $f_{Op}$ must be equal to the susceptance of a capacitor $C_{IN}$ that is needed to reach a match point.

2 $$\begin{aligned} \frac{jb}{Z_0}=j 2\pi f C_{IN}, \end{aligned}$$

so that

3 $$\begin{aligned} C_{IN}=\frac{b}{2\pi f Z_0}. \end{aligned}$$

Using this technique, an optimal value of $C_{IN}$ is calculated according to Eq. (3). This capacitor must be installed on the board next to the inductor’s input terminal to keep its close proximity to the calibration reference plane. Clearly, the added capacitor $C_{IN}$ must keep the same value and low loss within the entire temperature range. We use high Q/low ESR ceramic capacitors by Johanson Technology for which the experimentally observed variation of $C_{IN}<2$ % from 300 K to 3.6 K^13^ ensures the validity of this approach. The use of balancing capacitor $C_{IN}$ for reaching the matching conditions limits the range of impedances that can be matched with this method, yet the highly resistive types of DUTs discussed here fall within the range of acceptable impedances.

Let us consider the case $L=820$ nH for which the impedance trajectory lies within $r=1$ circle at room temperature, red arc in Fig. 4c. Importantly, at low temperature 3.6K the impedance trajectory expands out of it and crossing $g=1$ in the upper part the Smitch chart thus making it possible to find an appropriate positive value of $C_{IN}$. The addition of balancing capacitor $C_{IN}$ translates operating point very close to the middle of the Smith chart, i.e to the match point. From the data shown in Fig. 5b we calculated $C_{IN}\approx {26}$ pF. Optimization of $C_{IN}$ value performed in ADS software yields very similar result. To confirm the validity of our considerations we performed measurements using two close values of $C_{IN}$, 20 and 27 pF. The results are presented in Fig. 5b as two trajectories: red, which corresponds to $C_{IN}=20$ pF and blue, which corresponds to $C_{IN}=27$ pF. As expected, both capacitors result in a close match, with $C_{IN}=20$ pF case corresponding to a slightly overcoupled MN, and $C_{IN}=27$ pF to a slightly undercoupled MN. We need to point out that determination of balancing capacitor may represent a challenge if MN impedance trajectory for $C_{IN}$ expands very close to the edge of the Smith chart thus reducing the accuracy of $C_{IN}$ determination.

**Figure 6:**
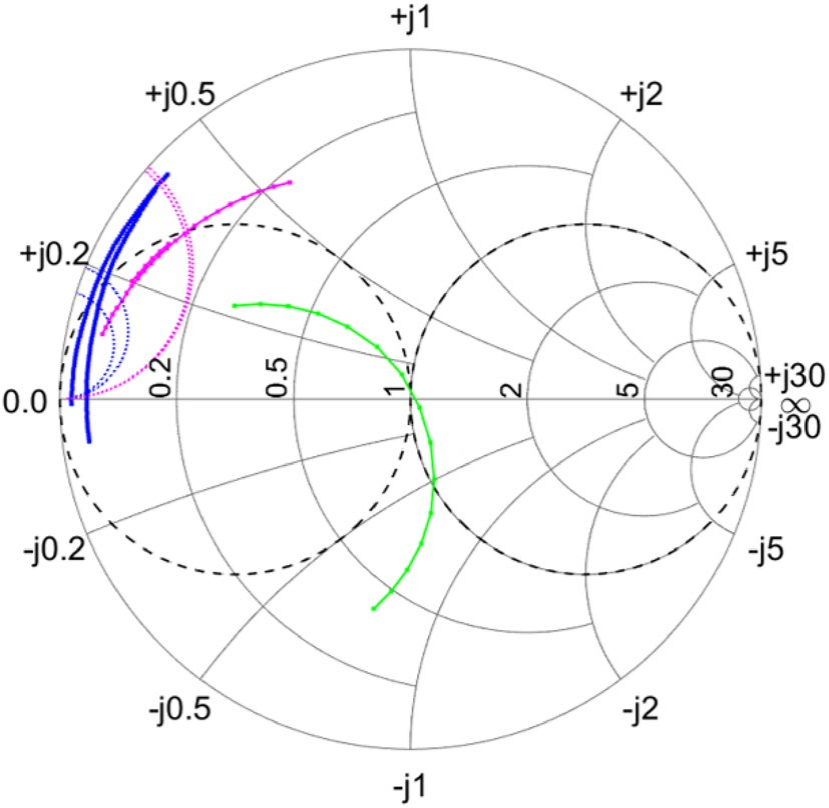
Smith chart showing impedance trajectories for ECD reflectometry measurements using two similar calibrations for MN composed of L = 240 nH inductor and $C_{IN}=$ 0 (blue dots), 15 pF (magenta dots), 30 pF (greeen dots). Imperfections in calibration results in uncertain definition of $C_{IN}$ needed to achieve good match for $C_{IN}=$ 0. A ”half-capacitance” guess (magenta dots) results in a much more accurate determination of $C_{IN}$.

Let us now proceed with the case where $L=$240 nH is connected in series with $C_{pad}$ = 467 fF (see Sect. S5 in the Supplementary materials on how it is extracted) and DUT connected in parallel to $C_{pad}$; note that all the experimental data except Fig. 4 are acquired with that DUT. The two blue traces in Fig. 6 that cross the $g=1$ circle there are experimentally obtained at very close temperatures 3.6K and 4K. However, calibration inaccuracy for heavily overcoupled MNs, when the impact of error correction on impedance trajectory is very significant, results in distinctly different impedance trajectories and two different constant susceptance curves (two blue dashed lines in Fig. 6) that determine $C_{IN}$. In addition to that, the low resolution of the Smith chart for impedance $Z_{IN}\approx 0$ makes it harder to determine the correct $C_{IN}$. The values obtained from the chart using Eq. (3) are in the range 39 to 45 pF. Therefore, for more accurate determination of $C_{IN}$ a smaller (e.g. 2 times smaller) than needed for match capacitor can be used to do a ”pre-match” experiment, so that the method from “Optimization of matching network” can still be applied but with far more accurate result. Indeed, in this case for $L=$ 240 nH by adding a small ”pre-match”capacitor $C_{IN}$=15 pF, the impedance trajectory changes (magenta dots, the results again obtained using two calibrations, 3.6K and 4K in Fig. 6 ), due to much better defined resonance. The impact of imperfect error correction is less significant, and both traces are overlapped. The crossing of the $g=1$ circle then results in two overlapping constant susceptance traces that cross the Smith chart edge with the suggested value of an extra 20 pF needed in addition to the existing 15 pF, thus making the suggested $C_{IN}=15+20\approx {35}$pF.

### Gate reflectometry: sensitivity and bandwidth tuning

The intent of the method explained above in “Optimization of matching network” is to achieve close to matching conditions in order to yield the best SNR. However, in experiment the optimal conditions for sensing may represent a trade off between the largest SNR and the bandwidth (BW) available to perform sensing using a DUT. Here we experimentally investigate various settings for MN to determine the optimal operating conditions and adjust it depending on the task of the sensing. For this experiment we use MN characterized in Fig. 6 (L = 240 nH, $C_{pad}$ = 467 fF) and utilized a SEBA composed of 200 nominally identical single-electron boxes as the DUT to conduct these measurements^12,13^

To map out the influence of the balancing capactior $C_{IN}$ on SNR and BW we perform six cycles of low-temperature measurements for $C_{IN}$ set at 0, 6.8 pF, 15 pF, 30 pF, 46.8 pf and 60 pF. For each experiment the selected $C_{IN}$ is soldered to the central line of the co-planar waveguide (CPW), like it is shown in Fig. S3 in the Supplementary materials, while keeping connection to the SEBA intact.

In each measurement cycle we first characterize the MN by sweeping the carrier frequency $f_{RF}$ and determine the optimal RF frequency $f_{Op}$ for a given $C_{IN}$ using error-corrected data to find a minimum of $\mid \Gamma \mid$. In this process we also model MN using parasitics of the inductor adjusted for low temperature and found very good agreement between the error-corrected data and simulations (see Sect. S5 in Supplementary materials for details). From the Smith charts in Fig. 6 it is clear that the closest to match point conditions are observed for $C_{IN}=$ 30 pF, while for larger $C_{IN}$ the DUT impedance $Z_{IN}$ is undercoupled ($Z_{IN}> Z_{0}$) and for smaller $C_{IN}$ it is overcoupled ($Z_{IN}< Z_{0}$) to the feedline.

**Figure 7:**
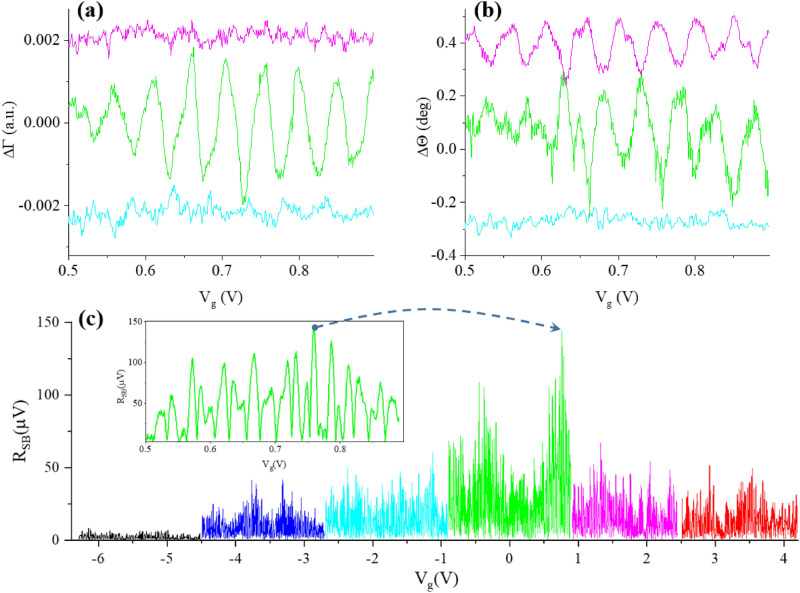
Coulomb blockade oscillations $Y(V_{g})$ of the SEBA measured as magnitude (**a**) and phase (**b**) of reflection at the operating frequency $f_{Op}$ using ECD for three values of the balancing capacitor $C_{IN}$: 15 pF (light blue), 30 pF (green), 45.8 pF (magenta). Constant values of magnitude and phase are subtracted from each curve to eliminate DC offset and curves are further vertically offset for clarity. Each trace is acquired with 40 ms time constant, no trace averaging is performed. No modulating signal is applied. (**c**) Comparison of SEBA oscillations $Y(V_{g})$ detected as SB magnitudes for modulating signal $\approx {0.07e}$ applied to the gate of SEBA obtained for six cases of $C_{IN}$: 0 pF (black); 6.8 pF (blue); 15 pF (light blue); 30 pF (Green); 45.6 pF (magenta); 60 pF (red). Curves are horizontally offset for clarity. T=3.6K. Inset: zoom into beating oscillation pattern for $C_{IN}$= 30 pF. The arrow points to the working point for $C_{IN}$= 30 pF, $V_{g}$ = 0.759V.

Once operating frequency is determined in each measurement cycle for each $C_{IN}$ by finding a minimum in an error-corrected value of $|\Gamma |$, we measure the magnitude of the demodulated signal as a function of gate voltage $V_{g}$ (no modulating signal is applied to the gate for this experiment). The oscillations of magnitude (a) and phase (b) of reflection coefficient in response to the variation of gate voltage applied to SEBA are plotted in Fig. 7 for three different values of $C_{IN}$ in the MN. These oscillations represent a beating pattern resulting from asynchronous single-electron charging of the SEBs in the array^12,13^. Since random offset charges are changing with every thermal cycle no close correlation in oscillations shape between thermal cycles is expected^13^.

The data shown in Fig. 7a,b are error corrected and therefore can be directly compared quantitatively. Note that the distribution of phase and magnitude components in the ”raw” (Fig. S9 in Supplementary materials) signal differs drastically from error-corrected data. Figure S9 shows that the raw data show very weak oscillations in magnitude of reflection $\Delta \Gamma (V_{g})$, and strong oscillations in phase $\Delta \Theta (V_{g})$, in a stark contrast with error-corrected data where oscillations are visible in both components. This example shows that without proper calibration the interpretation of the results based on raw data (e.g. a statement claiming that measured phase shift is solely due to a capacitive change in the $Z_{DUT}$) can be misleading.

As expected, the signal strength is greatly enhanced when a well-matched MN is used. It is important to note that for both cases away from matching the demodulated signal contains > 10 times larger DC component (i.e. DC offset independent on $V_{g}$ that must be subtracted in order to plot it in one figure, see Fig. S7 in Supplementary materials). This corresponds to the reduction of modulation depth produced by the Coulomb blockade oscillations in the SEBA in case of poor matching.

For more accurate quantitative evaluation of SNR and available bandwidth and their dependence on $C_{IN}$ we utilize the setup Fig. 2b with gate modulation signal, $\tilde{V_{g}}$, turned on. In this case a small sinusoidal signal $\tilde{V_{g}}$ at a frequency $f_{Mod} \ll f_{Op}$ is applied to the gate of the SEBA. The sweep of gate voltage $V_{g}$ modulates the admittance of SEBA^12,13^ which results in generation of sidebands (SBs) in the reflected signal $f_{Op} \pm f_{Mod}$^14^. Analysis of SB signals enables significantly more accurate evaluation of SNR and BW because it eliminates the DC background along with 1/*f* noise present in the demodulated carrier signal and instead performs synchronous detection of the signal in the narrow band ($\approx$ 1 Hz) near $f_{Op}\pm f_{Mod}$ defined by the demodulator time constant (30 ms).

An example of experimentally obtained SEBA oscillations in the SB magnitude acquired over $\pm 0.9\, V_{g}$ range (curves are horizontally offset for clarity) for six different values of $C_{IN}$ is presented in Fig. 7c.

The RMS amplitude of the modulating signal at $f_{Mod}$= 8 kHz is 3.5 mV, corresponding to an oscillating charge of $\approx {0.07e}$ applied to the gate of SEBA. The amplitude of modulating signal is chosen to operate within a linear part of the slope $d\mid \Gamma \mid /dV_{g}$ of the oscillation in Fig. 7a,b the by sweeping the magnitude of $\tilde{V_{g}}$ and making sure the magnitude of SB signal scales linearly with increase in $\tilde{V_{g}}$. Likewise, the amplitude of the carrier signal is selected to operate in the linear regime. This is achieved by sweeping the RF carrier amplitude and ensuring the magnitude of SBs scales linearly with increasing RF amplitude. The peak RF amplitude (${\widetilde{a}}$ in Fig. 2a) is then fixed at a level of 100 mV at the output of RF oscillator and attenuated by $\approx {60}\,\ dB$ with attenuators and directional coupler, which results in $\approx {70}\,\ dBm$ of power applied to MN. The reflected signal is amplified by $\approx {70}\,\ dB$ before reaching the input of the lock-in. In correlation with results obtained for $\Delta \mid \Gamma \mid$ signals (Fig. 7a,b), the magnitude of SB signals is very significantly boosted by appropriate choice of MN.

**Figure 8:**
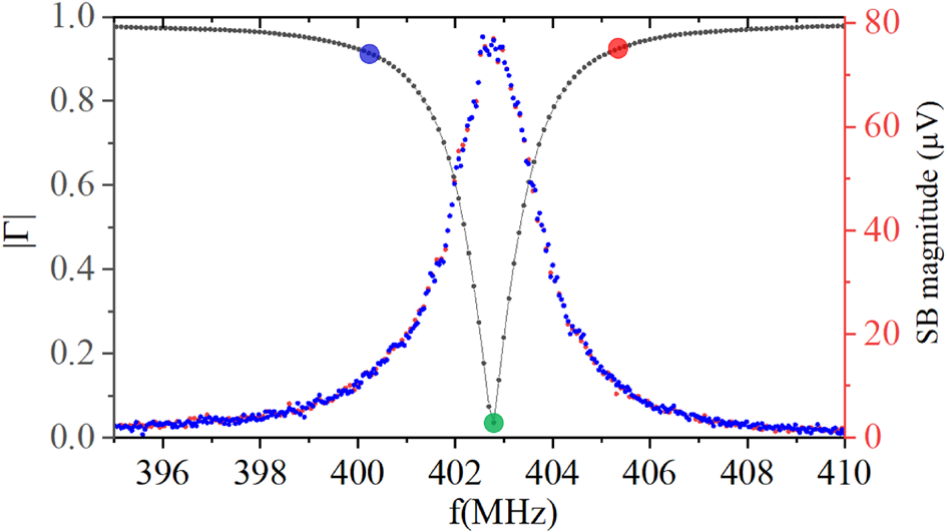
Error-corrected magnitude of reflection $\mid \Gamma \mid$ (in black ) and magnitudes of demodulated sidebands (lower SB -blue dots, upper SB - red dots) vs carrier frequency for MN connected to SEBA with $L=$240 nH and $C_{IN}$  = 30 pF. The signal is measured at the input of the UHFLIA in Fig. 2a. Sinusoidal signal $\tilde{V_{g}}$ at $f_{Mod}$= 8 kHz with RMS value of 3.5 mV is applied to the gate of SEBA along with a constant $V_{g0}$  = 0.879 V, positioning the response of the SEBA at the peak in SB signal in Fig. 7c. The three selected operation frequencies are shown by blue (400.6 MHz), green (402.6 MHz) and red (404.6 MHz) dots. T=3.6K.

In fact, the magnitude of SB for $C_{IN}=30 pF$ is scaled up by a factor > 15 compared to $C_{IN}=0 pF$. Direct acquisition of SB signals also allows optimization of the operating carrier frequency by finding the maximum of the magnitude of SB signals. Fig. 8 shows a frequency response of MN, $\mid \tilde{\Gamma }\mid (f_{carrier})$ (black dots) and both upper (red dots) and lower SB (blue dots) for low frequency modulation applied to the gate. As expected, the maxima in SB are in perfect correlation with the minimum in the magnitude of reflected carrier signal.

**Figure 9:**
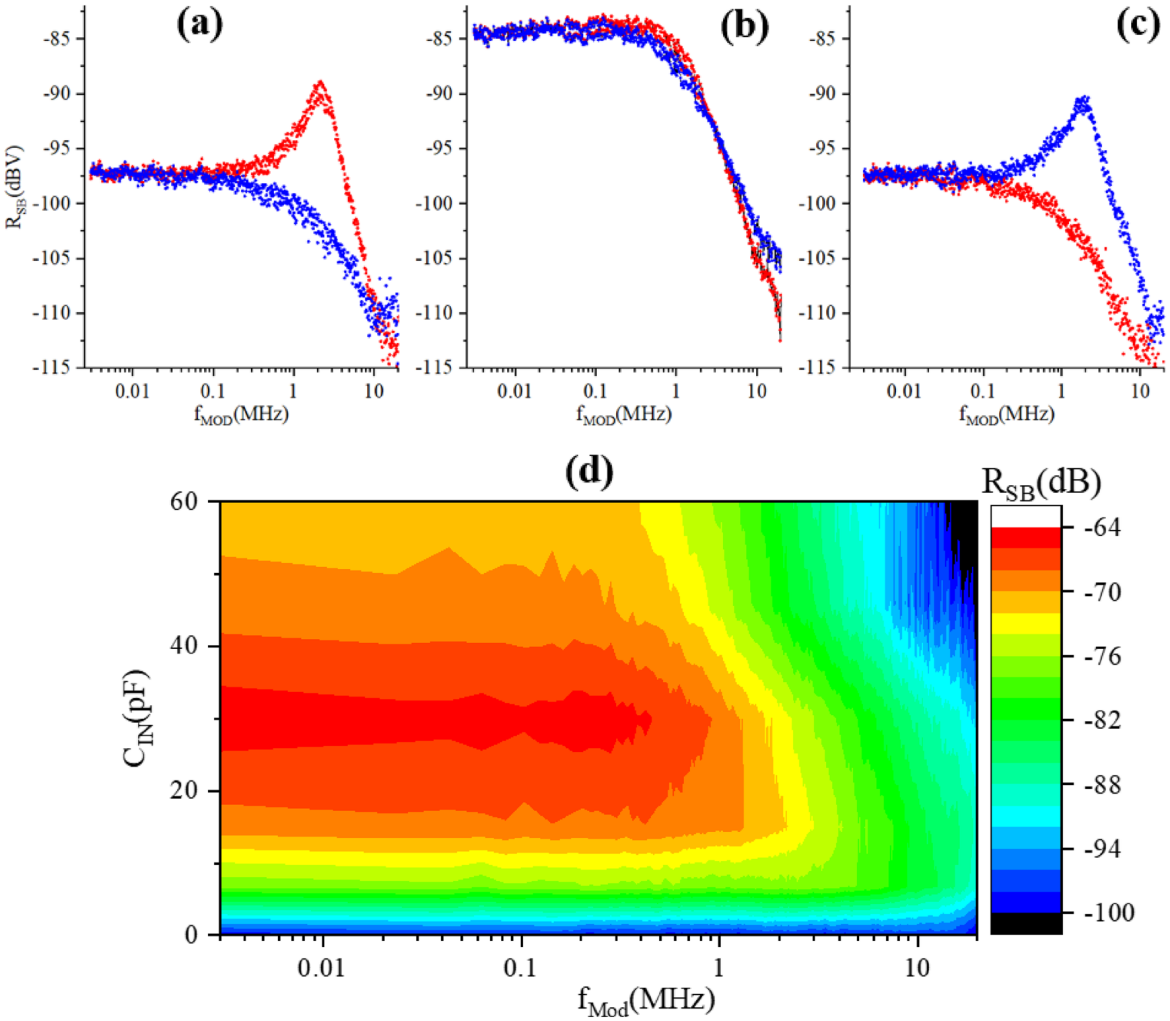
RMS value of upper (red) and lower(blue) SB vs modulation frequency $f_{Mod}$, $\tilde{V_{g}}$=3.5 mV RMS measured at the input of UHFLIA using setup Fig. 2a; for well-matched case $C_{IN}=$30 pF with resonant frequency of 402.608 MHz. Subplots a-c correspond to different carrier frequencies 400.6 MHz (**a**); 402.6 MHz (**b**); 404.6 MHz (**c**), marked by dots in Fig 8. (**d**) Color map showing experimentally obtained relative SB magnitude (in dB) vs frequency of modulating sinusoidal signal $\tilde{V_{g}}$ applied to the gate of SEBA for chosen input $C_{IN}$. Carrier frequency for each $C_{IN}$ is adjusted to obtain maximal SB magnitude at low modulation frequency $f_{Mod}<10$ kHz (see Fig. S8b in Supplementary materials for the exact values of carrier frequency). Color represents normalized magnitude of SB. For normalization the carrier signal level out of resonance 395 MHz and 410 MHz in Fig. 8 for $C_{IN}=$30 pF is taken as 0 dB. Temperature of experiment is 3.6 K.

Once SEBA oscillations in SB are characterized and optimal operation frequency $f_{Op}$ is found, for each value of $C_{IN}$ the strongest peak in demodulated SB signal $R_{SB} (V_{g}$) is identified at some $V_{g}$ = $V_{g0}$ within $\pm 0.9 V_{g}$ span (inset in Fig. 7c)), and the bandwidth available with this setting of $C_{IN}$ for SEBA sensing is characterized at this $V_{g0}$. For that purpose the frequency response of SEBA to the modulation frequency $f_{Mod}$ is measured using setup Fig. 2a by acquiring SB signals as we sweep modulation frequency $f_{Mod}$ in the range 1 kHz–20 MHz for each value of $C_{IN}$. It is important to note that the carrier frequency needs to be chosen appropriately for each setting of $C_{IN}$. Figure 9a–c shows frequency response of the SEBA in the upper and lower SB measured for three different settings of carrier frequency. If carrier frequency $f_{RF}$ is chosen 2 MHz below (above) the optimal $f_{Op}=402.608$ MHz, the resulting SB signals are greatly attenuated at all modulation frequencies except near $f_{RF}= f_{Op}+f_{Mod}$ for the lower SB, subplot Fig. 9c (or near $f_{Op}-f_{Mod}$ for the upper SB, subplot Fig. 9a ), when the resulting tone lands in the pass-band near $f_{Op}$. When $f_{Op}$ is chosen at the minimum of $\Gamma$ (obtained using ECD protocol), the magnitude of both sidebands stays constant at $\approx {-85}$ dBV level within $\approx {1}$ MHz until the -3 dB level is reached at $f_{Mod}\approx {1.2}$ MHz Fig. 9b. Clearly, the choice of appropriate carrier frequency, $f_{RF}= f_{Op}$, yields the strongest response within the entire pass-band.

These measurements combined together enable direct comparison of the magnitude of SB signals generated at different $C_{IN}$ settings over the sensing BW of interest. Figure 9d provides a composite 2D picture for tuning of the MN with balancing capacitor $C_{IN}$. Here, the color-coded strength of the relative SB magnitude referred to applied RF signal (in dB) in response to modulating signal is plotted vs modulating frequency $f_{Mod}$ (X axis) and balancing capacitor $C_{IN}$ (Y axis). One can see that for $C_{IN}$ = 0, when the matching network is out of tune, the relative magnitude of the generated side band signal is the smallest: the signal (blue) is $\approx$ − 97 dB, i.e. about 24 dB weaker compared to well-matched case (red), where is reaches $\approx$ − 64 dB. As the value of $C_{IN}$ increases from 0 to $C_{IN}$  = 30 pF ($\approx C_{match}$), closest to the matching conditions, the strength of the SEBA response in the pass-band also increases, but at the same time its bandwidth shrinks. Further increase of $C_{IN}$ beyond $C_{match}$ monotonically reduces the magnitude of the gate response and it continues to reduce the available bandwidth. Therefore, by using $C_{IN}=C_{match}$ one achieves the best trade-off between sensitivity and bandwidth. These experimental results are in good qualitative correlation with the simulations (see supplementary information, plot S9). To perform simulations of the sideband signal the response of SEBA admittance to the input $\bar{\bar{V}}_{g}$ is first simulated and the working point is defined (see part S5 in the Supplementary information for details). Next, the admittance equivalent of SEBA is connected to the MN simulated using optimized inductor model and its response to the modulating signal, ${\tilde{V}}_{g}$ = 3.5 mV RMS, is calculated for $f_{Mod}$ in the frequency range, 1 kHz–20 MHz (see part S6 in the Supplementary information for details).

Note that error-corrected data in this work are only available for the carrier frequency signal because CEC protocol is only performed for demodulated $f_{RF}$ which is schematically represented a [e]^−1^ box in Fig. 2a. This precludes a direct numerical comparison of experimentally acquired side-band signals with simulations, except perhaps the case where MN is close to matching and error-correction is minimal. Nonetheless simulations provide a good way to look at the performance of SEBA connected to MN in terms of magnitude and bandwidth of generated sidebands. In our future work we plan to embed CEC protocol into SB demodulation path using Zurich instruments software^27^.

**Figure 10:**
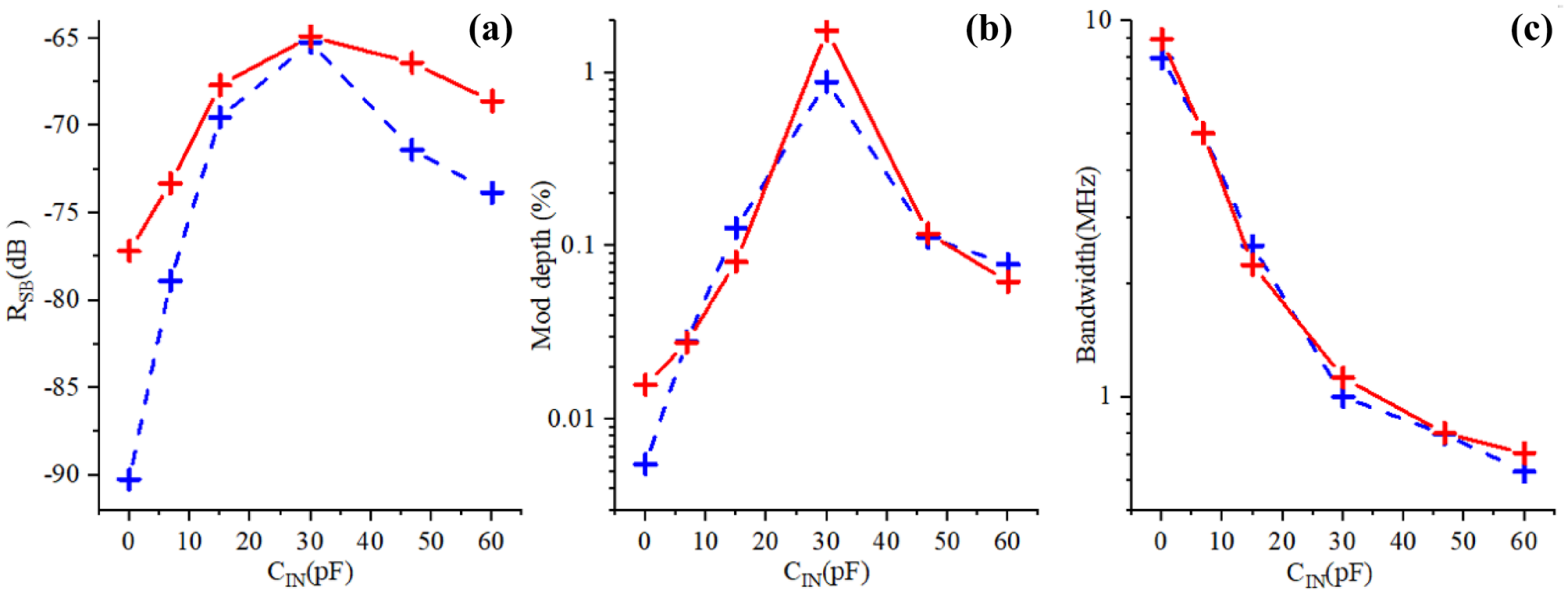
Three important parameters plotted vs balancing capacitor $C_{IN}$. Red crosses with solid lines—simulation, blue crosses with dased lines-experiment. Left: SB magnitude normalized to $C_{IN}=30$ pF case. Middle: Modulation depth, in percent; ${\tilde{V}}_{g}=$3.5 mV at $f_{Mod}=$ 8 kHz. RF carrier magnitude before attenuation is 65 mV peak for all $C_{IN}$, except for $C_{IN}=$ 0 where it is 300 mV peak to account for low SNR. Right: Bandwidth (on -3 dB level) $f_{Mod}$ available for SEBA sensing. Note that here the experimental data are not subjected to calibration/error correction.

Therefore in this work direct quantitative comparison between experiment and calculation cannot be performed for SB and only qualitative analysis is possible. Indeed, the simulations assume an ideal network analyzer, while in experiment the performance of MN, particularly for $C_{IN}$ close to zero suffers from various non-idealities, e.g. destructive interference in the standing waves in the cables. Therefore, simulations underestimate the loss of signal away from the match point. This is clearly illustrated in Fig. 10a where simulations predict significantly smaller signal degradation for $C_{IN}$ approaching zero.

The experimentally obtained and simulated modulation depth, defined as a ratio of *SB*/*Carrier* magnitudes, and expressed in percents, is plotted vs $C_{IN}$ in Fig. 10b. Experiment and simulations are in good correlation, except for small values of $C_{IN}$ where simulated modulation depth overestimates the performance due to assumed ideal reflectometer. In either case the modulation depth is very low for $C_{IN}=$0 ($\approx 10^{-2}\%$), it reaches maximum of $\approx {1}\%$ for $C_{IN}=30$ pF, for closest to match conditions and then goes down again but at a less steep pace for balancing capacitor values that exceed the optimal. Very small modulation depth implies very inefficient energy transfer to and from DUT, and ultimately reduces the dynamic range for measurements of $|\Gamma |$ due to a large constant (i.e. $V_{g}$ independent) value after demodulation. Finally, Fig. 10c shows very good correlation between experiment and simulations for the BW accessible for SEBA sensing.

The results presented in Fig. 10 clearly demonstrate that the sensitivity drops abruptly for $C_{IN} \ll C_{match}$ but enables a broader flat portion of available bandwidth while for $C_{IN} > C_{ match}$ the monotonic drop in sensitivity is accompanied by a significant loss of bandwidth. Here $C_{match}$ is the optimal value of $C_{IN}$ which provides closest to match settings of MN.

## Conclusions

We propose and demonstrate a simple two-step method for optimizing $\Pi$ MNs for low temperature reflectometry of large ($\gg 1\hbox {M}\Omega$) DUT impedances. By performing a measurement of the partial MN utilizing an inductor *L* with unknown parasitics and capacitance $C_{pad}$ in parallel to a DUT, a balancing capacitor $C_{IN}$ can be found as long as the partially assembled MN with attached DUT represents a load overcoupled to the feed line, the condition fulfilled for nearly all studied inductors of the 0805LS series^15^. This method requires a low temperature calibration of the reflectometer in order to remove effects of non-idealities in the reflectometer, and leads to more numerically meaningful measurements. Performing a low temperature calibration is a time-consuming process requiring multiple thermal cycles of a cryostat. Because of this, there is inevitable error present in the error correction model that ends up in the final error corrected data. However, these errors are small enough that the error corrected data can still be used to produce meaningful results.

Experiment also shows that to achieve quantitative accuracy the simulations performed to model matching networks must take into consideration parasitic components of the inductors used in MN^12,13^, and neglecting these parasitics leads to significant errors in the evaluation of sensitivity and bandwidth. We compare the simulations results of two models (see part S7 in supplementary materials for more details). Model A used in this work and^12,13^ takes into account low-temperature values of parasitic components while model B^1,3^ utilizes an ideal inductor and a leakage resistance $R_{d}$ in parallel to $C_{pad}$ introduced to account for experimentally observed losses revealed by a presence of a dip in the magnitude of reflection coefficient $\Gamma$ . Figure S11 in Supplementary materials confirms the validity of approach used in this work.

Our measurements demonstrate that achieving a good match can have a very significant impact on the ability to measure weak signals. This is clearly demonstrated by performing a detailed comparison of the SB magnitudes for different settings of $\Pi$ MN tuned by a balancing capacitor $C_{IN}$: the SB signal strength is greatly enhanced when the close to match conditions are achieved. Experiments have shown that the SB signal enhancement exceeds 20 dB compared to “as is” MN with $C_{IN}$ = 0. The underlying physical reason for such improvement is that by achieving close to match conditions we achieve the most efficient energy transfer when the load to the feedline is closest to $Z_{0}$. As a result, the negative impacts of various non-idealities in this situation are canceled to a large degree in contrast with grossly mismatched conditions where these non-idealities such as destructive interference only exacerbate the existing mismatch.

Both experiment and simulations show that the presence of balancing capacitor $C_{IN}$ shapes the bandwidth available for DUT operation and BW is monotonically reduced with the increase in $C_{IN}$. By contrast, the magnitude of detected SB increases rapidly for $C_{IN}<\,C_{match}$, and slowly decreases for $C_{IN}>\,C_{match}$.

## Supplementary Information

Supplementary Information.

## Data Availability

The data that support the findings of this study are available from the corresponding author upon reasonable request.
