# Supplementary material for: Gate reflectometry of single-electron box arrays using calibrated low temperature matching networks
Source: Sci Rep. 2022 Feb 23;12:3098. doi: 10.1038/s41598-022-06727-1 (PMC8866512; doi:10.1038/s41598-022-06727-1)
Supplement: Supplementary file 1 — Supplementary Information. [file 41598_2022_6727_MOESM1_ESM.pdf]

Supplementary information.
Gate reflectometry of single-electron box arrays using calibrated low temperature matching networks

Matthew J. Filmer[1,2*], Matthew Huebner[1], Thomas A. Zirkle[1,3,+], Xavier Jehl[4,+], the late Marc Sanquer[4,+], Jonathan D. Chisum[1,+], Alexei O. Orlov[1,+], and Gregory L. Snider[1,+]

[1]Department of Electrical Engineering, University of Notre Dame, Notre Dame, IN 46556, USA
[2]L3Harris, Fort Wayne, IN 46818, USA
[3]Northrop Grumman, Ogden, UT 84405, USA
[4]PHELIQS-LATEQS, Université Grenoble-Alpes and CEA-Grenoble, Grenoble 38054, France
[*]corresponding author mfilmer@alumni.nd.edu

1 Calibration and Error correction protocol

The systematic errors due to cables, connectors, and all other linear non-idealities of the measurement system can be represented in a circuit model with the introduction of an "error box" (EB), so that the real instrument (in our case, UHF ZI Lock-in) is replaced with an ideal instrument and EB. The EB is simply a 2-port network whose behavior encapsulates all the errors of the system, as shown in Figure S1. By performing an error calibration, these non-idealities can be quantified and mathematically removed from subsequent measurements. In doing so, it allows measurements to be compared quantitatively against measurements taken on other systems, or against circuit models. For SOL calibration, as the name implies, the three impedances chosen are *Short* (Z=0 Ω), *Open* ($Z = \infty\,\Omega$), and a matched Load ($Z = Z_0$=50 Ω). Any three different impedances can be used, however these standard values are a good choice as they cover a large area of the impedance space, increasing the accuracy of the calibration, and are easy to fabricate. To perform a calibration, a reflection measurement at the frequency band of interest is taken for each standard. These will be referred to as the measured reflection given by $\Gamma_{MS}, \Gamma_{MO}, \Gamma_{ML}$, for the *Short*, *Open*, and *Load*, respectively. These values, along with the expected reflection of each standard ($\Gamma_S = -1, \Gamma_O = 1, \Gamma_L$=0) are used to calculate three of the S-parameters of the error box ($e_{11}, e_{21}e_{12}, e_{22}$). Once these error parameters are known, it is simple to de-embed the error box from measurements. The errors are removed from the measured reflection using

$$\Gamma = \frac{\Gamma_M - e_{11}}{\Gamma_M e_{22} - e_{11}e_{22} + e_{21}e_{12}} \tag{1}$$

Here Γ_M, is the measured reflection of a DUT and Γ is the error corrected reflection. To compute the error-corrected data (ECD) from raw data a simple Matlab script is used to remove the errors from the measured reflection using equation 1. A useful side

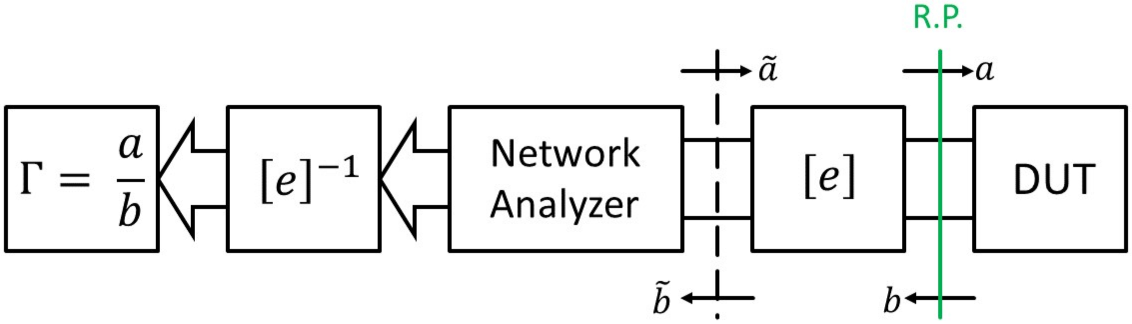

Figure S1. Error correction protocol. R.P. is the reference plane at which calibration is performed. In our experiments ZI UHF is used as a network analyzer

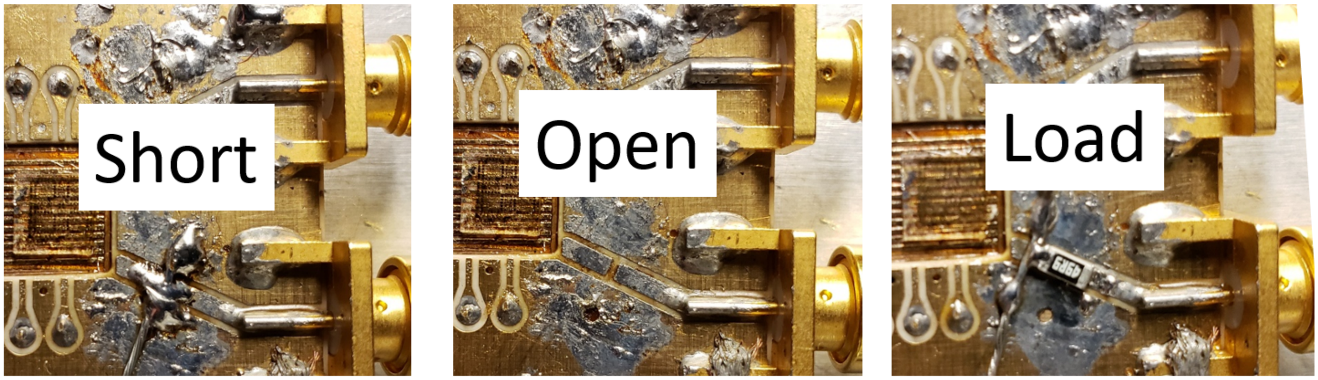

Figure S2. SOL kit used in the experiments

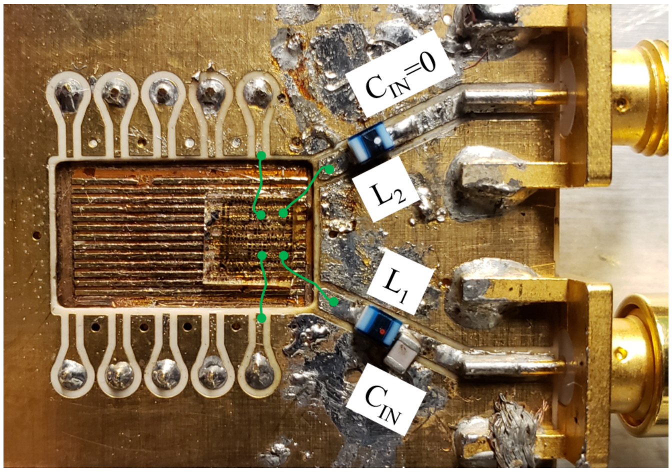

Figure S3. Printed circuit board used in the experiments. Two SEB arrays are tested simultaneously with 2 channels of ZI UHF (L1=240 nH, L2=270 nH)

effect of performing these calibrations is that the gain or attenuation of all the components within the signal path need not be known. All of these will be a part of the extracted error terms and will therefore be automatically corrected when performing an error correction. This is helpful since the precise behavior of these components often varies with frequency and is not precisely characterized. An error correction removes the need to precisely characterize each individual component in the signal path, as the error calibration does it all at once as long as the components remain in their linear regimes.

A collection of three known terminations are assembled and measured on a printed circuit board identical to the actual board containing MN elements and devices in three successive thermal cycles within 3 days and error corrections are computed (Fig. S2). (Figure S2b). For the *Short* a piece of copper braid was placed atop of the input pad of the inductor and soldered to the ground plane. To represent the *Open* the space for inductor is left empty on the same piece of coplanar waveguide; the DC resistance of *Open* is in excess of 1 GΩ. For the *Load* a 49.9 Ω resistor soldered to the PCB trace of coplanar waveguide is installed in place of the inductor with the same chip size while at the other end the resistor is grounded . The four probe low frequency resistance of the *Load* is measured using a lock-in amplifier and is found to vary by less than 0.1Ω in the range from 300 K to 3.6 K. For both terminators, it is assumed the impedance is purely resistive, and frequency independent within 1 GHz bandwidth[1]. The measured resistance of $R_{Short} < 0.02\,\Omega$ within the 300 K to 3.6 K temperature range.

2 Matching network and DUT assembled on a PCB

An example of two SEBA wire-bonded to two MNs on a PCB is presented in Fig. S3 with components of MNs labeled. RF circuit is assembled on a Rogers RO4350B high frequency substrate with a 240 nH and 270 nH Coilcraft 0805CS ceramic core surface mount inductors soldered to a coplanar waveguide (CPW). In the photo a capacitor C_{IN} is soldered next to the inductor L_1 is clearly visible. Bonding wires (in green) are sketched for visibility.

3 Results of error correction

An example of raw vs ECD measurements is presented in Fig. S4(a, b). Black curves represent raw data, and red dotted lines correspond to ECD in Fig. S4a-c. Note that multiple ripples in magnitude response and monotonic linear slope in phase response are eliminated after error correction which makes it very easy to identify the minimum in Γ suitable for reflectometry measurements.

4 Simulation of SEBA admittance

To simulate the gate dependent complex admittance $Y(V_g)$ for a SEBA in accordance with the model[2] the respective components $C_{dyn}(V_g)$ and $R_{Sis}(V_g)$ are calculated individually for each SEB for a frequency at which reflectometry measurements are performed ($\approx$ 400 MHz) and then summed together. To account for random offset charges the phase of the Coulomb blockade oscillations in each SEB is set purely random[3], while gate (C_g=3 aF) and junction (C_J=50 aF) capacitances are distributed with a standard deviation of 3% and 20% respectively.

The resistance of the junctions is assumed to be inversely proportional to the junction overlap area[4] so that

$$R_J = 100\,\mathrm{k}\Omega \left(\frac{50aF}{C_J} \right) \tag{2}$$

After the admittances of all (N=200) SEBs are summed together the resulting SEBA admittance curve over a span of a V_g sweep of 2V is plotted (Fig. S5). Next, the largest peak in admittance with the steepest slope within this V_g span was identified and approximated with two vertically offset sinewaves to represent the real and imaginary portion of SEBA admittance in the vicinity of this peak (inset in Fig. S5). This approximation is then used to model the gate dependent admittance of the SEBA for calculations of bandwidth and sensitivity in the vicinity of the working point positioned in the middle of the rising slopes of the $Re(V_g)$ and $Im(V_g)$ peaks. Note that the resulting average capacitance of the SEBA is on the order of 40 aF with the oscillatory part of it on the order of $\pm$6 aF.

5 Low temperature MN optimization

The purpose of the Π matching network in this target application (Fig. S6) is to tweak the value of C_{IN} to achieve a close match, i.e. to convert the complex DUT impedance $Z_{DUT}(V_g) = 1/Y(V_g)$ from tens of MΩ to $Z_{IN}(V_g)$ closer to Z_0 that would result in significant changes in $\Gamma = (Z_{IN} - Z_0)/(Z_{IN} + Z_0)$ and thus will boost the magnitude of the parameter of interest, $\Delta\Gamma(V_g)$. In order to match the experimentally observed low temperature characteristics of the MN for varying values of C_{IN}, the Keysight Technologies Advanced Design System (ADS) optimization toolkit is used. As a starting point for modeling, we use the Coilcraft model[5] with scaled down values of DC and skin-effect resistance; the values of C_1 and R_1 (associated with losses in the core) are set as free parameters, and inductance is allowed to change within 5 % of the nominal value. Here we take into account the, experimentally observed by Zirkle[4], reduction of an inductor's DC resistance is caused by reduction in the resistivity of copper coil, by a factor of $\approx$ 70 from 300K down to $\approx$ 10 K and associated reduction of skin-effect resistance proportional to the square root of resistivity $\approx$ 8 is used as a starting point for optimization. An experiment by Zirkle[4] also revealed very weak temperature dependence of the value of the capacitances C_{IN} (less than 2% for temperature variation from 300K to 3K) used in this work (0805 size surface mount High Q/low ESR ceramic capacitors by Johanson Technology). An

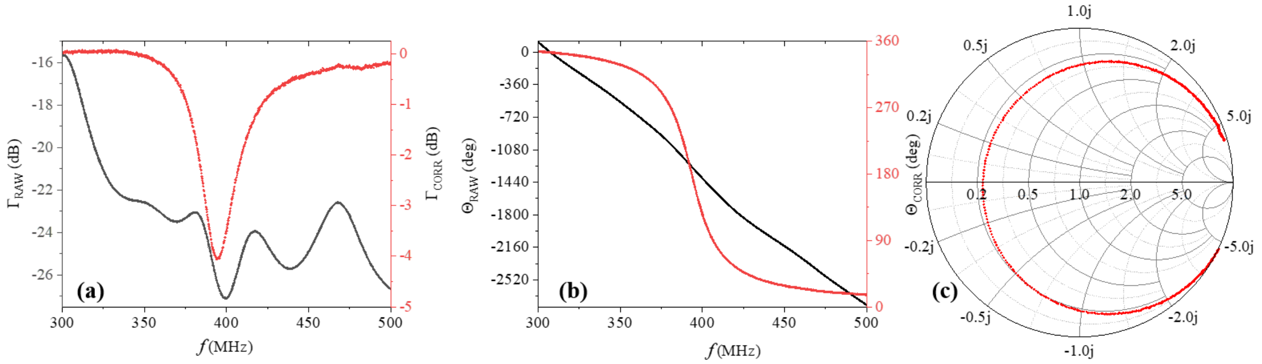

Figure S4. Results of reflectometry measurements without (black dots) and with error correction (red dots) at T=300K for a MN composed of L=240 nH inductor and C_{pad} (a) -magnitude, (b)- phase, (c) -Smith chart.

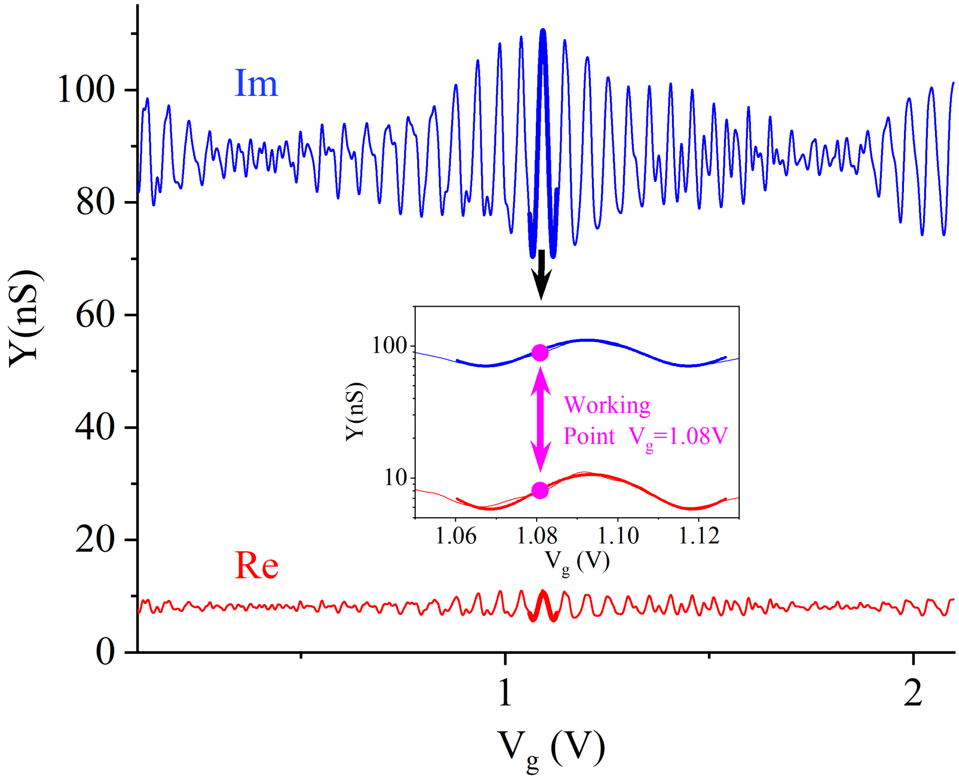

Figure S5. Simulated oscilations of $Re(V_g)$ (red) and $Im(V_g)$ (blue) parts of admittance $Y(V_g)$ of an array of 200 SEBs. Zoomed in area shows sinusoidal approximation of both components (thick lines) and a working point at which bandwidth and SB magnitude are simulated

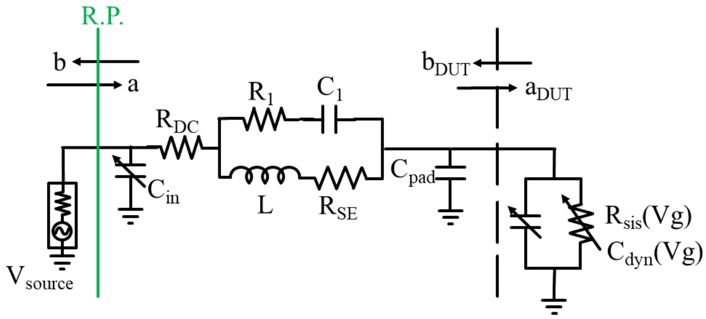

Figure S6. The circuit used for modeling of bandwidth and sensitivity of SEBA connected to MN with varying C_{in}

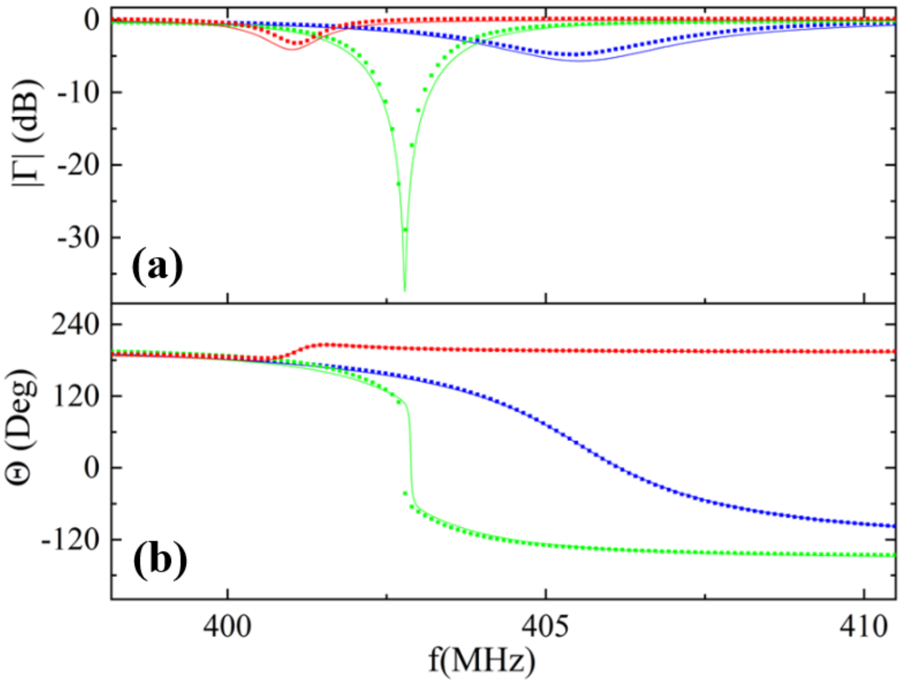

Figure S7. Experimentally measured error-corrected magnitude and phase responses of MNs with three different values of C_{IN} 15 (blue),30 (green),60 (red) pF and respective simulations using model shown in Fig S2. The following parameters are used in simulations $L = 247.9$ nH , $C_{pad} = 467$fF (for $C_1 = 169.7$ fF $R_{DC} = .0124$ Ohms and $K_{SE} = 1.9e-5$ where $R_{SE} = K_{SE} * \sqrt{F_{Res}}$). We also allow for small variations of C_{pad} (≤ 5 fF) based on potential movements of the bond wires between cooling cycles, C_{IN} is tweaked within its tolerance range of 5%

ADS hybrid optimization algorithm is used to find an equivalent circuit which best fits the experimental data. The cost function is the difference between $|\Gamma|$ of the measured response (after calibration) and $|\Gamma|$ of the simulated response and the fitted model parameters are C_1, R_1, and the value of inductance. The optimization is complete when the cost function is below the target of 0.1. The average value of pad capacitance obtained by the optimization is C_{pad} =467 ± 5 fF. Note that the C_{pad} is at least 10^3 times larger than the total capacitance of the SEBA connected in parallel.

The resulting characteristics of MN for the three values of C_{IN} : smaller than (blue) -, close to (green) -, and greater than (red) required for matching are shown in Fig. S7, which confirms a very strong correlation between the experiment and the model.

Fig S8 shows a comparison between experimental and simulated data for the magnitude of reflection coefficient $|\Gamma|$ (matching conditions must result in minimal reflection) and resonant frequency f_{Res} vs the value of a balancing capacitor C_{IN}. Good correlation between experiment and simulations is observed thus confirming the validity of the used model.

Fig. S9 shows a difference between the raw and error-corrected response of the SEBA, $\Delta \mid \Gamma \mid (V_g)$ (a) and phase $\Delta\Theta(V_g)$(b), respectively to a change in gate voltage V_g for the three different values of balancing capacitor C_{IN}. While both raw and error-corrected data are the strongest for a well-matched case (green curves) as a result of applying the CEC protocol the appearance of oscillations in magnitude and phase of reflection coefficient changes very significantly. Namely, it shows that the oscillations in SEBA admittance lead to oscillations in both components of the reflected signal.

6 Bandwidth and SB magnitude dependence on C_{IN}

To calculate the bandwidth available for the target application (i.e. fast voltage gate sensing with a SEBA) , the RMS magnitude of the sideband generated in response to the small sinusoidal signal gate modulation voltage $\tilde{V}_g$ at frequency f_{Mod} is computed. The SEBA is modelled as an oscillating complex admittance $Y(V_g)$ composed of its real $Re(V_g)$ and imaginary $Im(V_g)$ parts (see section S4 for details). Modulation signal magnitude $\tilde{V}_g$ =3.5 mV RMS is chosen to be small compared to ΔV_g =50 mV, the characteristic period of Coulomb oscillations in the SEBA. The working DC bias point ($V_g \approx 1.08V$) is shown in Fig. S5. The calculation of sideband magnitude is achieved through a harmonic balance simulation in ADS. This simulation models the SEBA's response to gate modulation after the carrier frequency is set to the MN resonant frequency for each value of C_{IN}

(Fig. S8b).

The relative sensitivity of the SEBA is expressed as a ratio, in dB, between the generated single sideband and applied RF carrier voltage for a chosen $\tilde{V}_g$ of 3.5 mV RMS. To calculate the bandwidth for SEBA sensing of $\tilde{V}_g$, the resulting magnitude of the sideband is graphed versus the modulation frequency. From here the bandwidth is calculated at a -3 dB level below the low frequency portion of the graph (see Fig. 9c in the main text).

We combine calculations of bandwidth and sideband magnitude for a MN composed of L=240 nH, C_{pad} = 467 fF and various settings of C_{IN}, from 0 to 60 pF in the 2D colormap shown in Fig. S10. Note that simulated data underestimate the losses of signal away from the match point ($C_{IN} \approx 32pF$) that occur in experiment, in particular near C_{IN} = 0 pF.

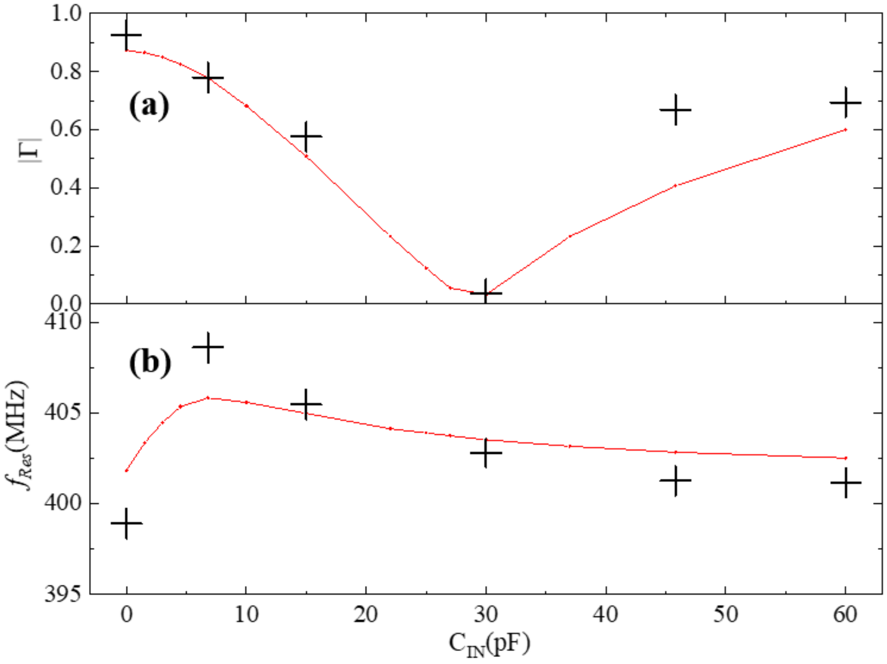

Figure S8. Calculated and experimentally measured ECD magnitude of reflection coefficient $|\Gamma|$ (a) and resonant frequency (b) using model in Fig. S7 for different values of C_{IN}. Black crosses -experiment, red dots with dashed red lines -calculations

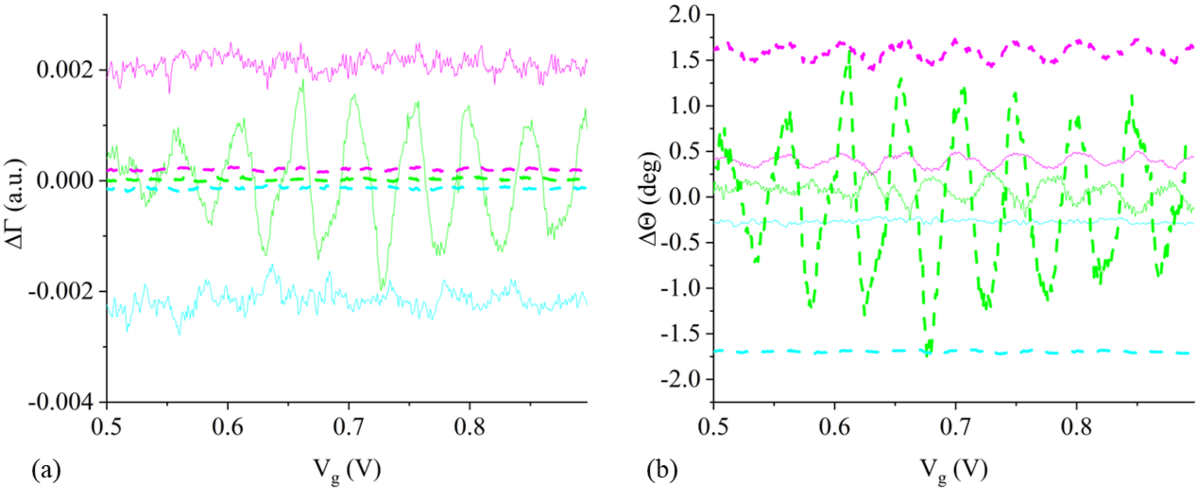

Figure S9. Comparison between raw (dashed lines) and error-corrected (solid lines) SEBA response to a gate voltage V_g sweep for magnitude $\Delta|\Gamma|(V_g)$ (a) and phase $\Delta\Theta(V_g)$ (b) of a reflected signal measured for three values of C_{IN}: 15 pF (light blue); 30 pF (green); 45.6 pF (magenta). The data are vertically offset for clarity.

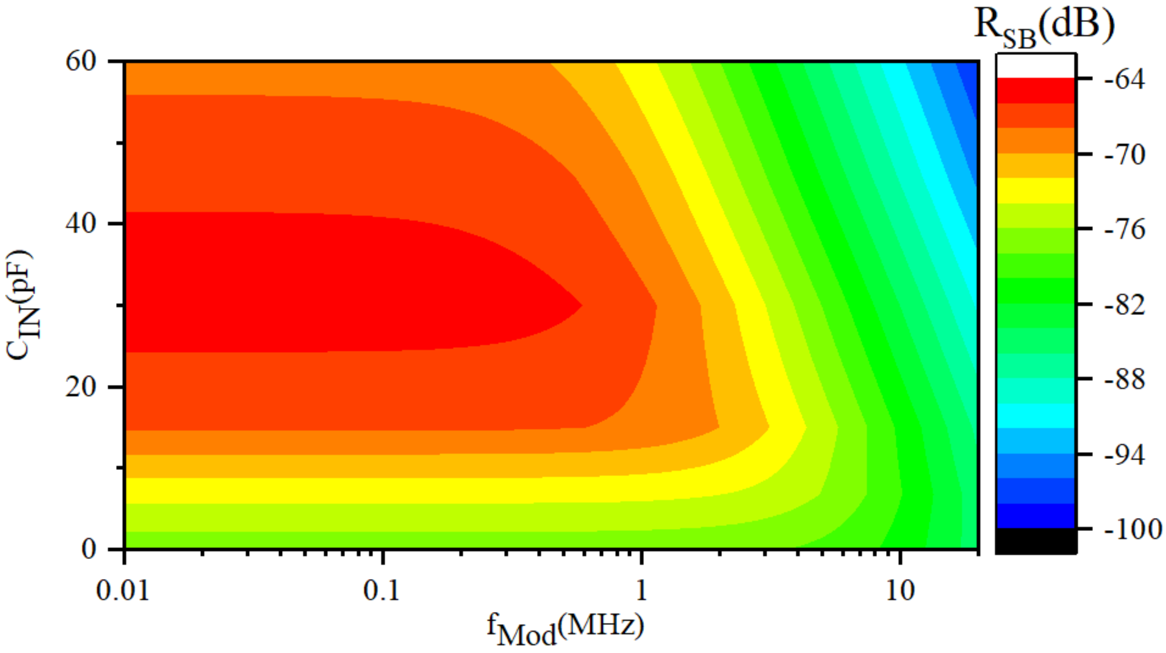

Figure S10. Simulated colormap showing sensitivity and bandwidth change for varying input capacitance C_{in} for the case of the SEBA discussed in section S4. Color represents the strength of an upper/lower sideband of the reflected signal modulated by an AC signal applied to the gate of the SEBA shown in Fig S1 @3.5K

7 Gate reflectometry model comparison

In several recent publications devoted to gate reflectometry[6,7] the inductor used in the MN is treated as an ideal element with no parasitic components and to account for the experimentally observed losses, revealed by a presence of a dip in the magnitude of reflection coefficient Γ, a leakage resistor to ground parallel to the DUT, R_d, is added to a model used by Gonzalez[6] and Ibberson[7]. Below we show that while providing a qualitative guidance, this approach results in significant errors when it comes to quantitative evaluation of sensitivity and bandwidth of the resulting MN.

To illustrate the difference resulting from the two models, A - used in this work and B - used by Gonzalez[6] and Ibberson[7] we perform the following simulations. For model A we use the circuit shown in Fig. S6 with a negligible dielectric loss to ground where inductor L=247 nH with parasitic components experimentally determined at low temperature is connected to a pad with capacitance C_{pad} =467 fF and DUT in parallel with C_{pad}. For model B we use an ideal inductor of the same value connected to a C_{pad} in parallel with the same DUT as in model A, and, following[7], assume an additional resistive loss to ground parallel to DUT, R_d= 30kΩ.

First we calculate matching conditions for both models, and next we evaluate the sensitivity to DUT admittance modulation by calculating a magnitude of the generated side band signal following the method described in section S6.

One immediate consequence of neglecting parasitic parallel capacitance of the inductor in model B (C_1 in Fig. S6) is the shift of resonance from ≈ 403 MHz to ≈ 470 MHz assuming the same pad capacitance, C_{pad}. To account for the experimentally observed resonant frequency within model B, the pad capacitance must be unjustifiably increased to C_{pad} = 654 fF. To achieve matching conditions, model A predicts C_{IN}=30 pF, in close correlation with the experiment while model B suggests using a smaller value of C_{IN}=14 pF. In our experiment, a very similar value, C_{IN}=15 pF, was tested and has proven to be too small to achieve a good match (see Fig S7-S9)

Next, we compare the predicted sensitivity to gate modulation for both models. For C_{IN}=30 pF model A predicts a bandwidth of 1.2 MHz and a relative sensitivity of -65 dB. By contrast, model B predicts much lower sensitivity, -82 dB, accompanied by a much broader bandwidth (about 6 MHz). The reason for this is fairly straightforward: the addition of unrealistically small shunt resistance R_d along with a larger value of C_{pad} reduces the signal from the DUT, and at the expense of sensitivity it widens the bandwidth. Experimental results again confirm the validity of model A.

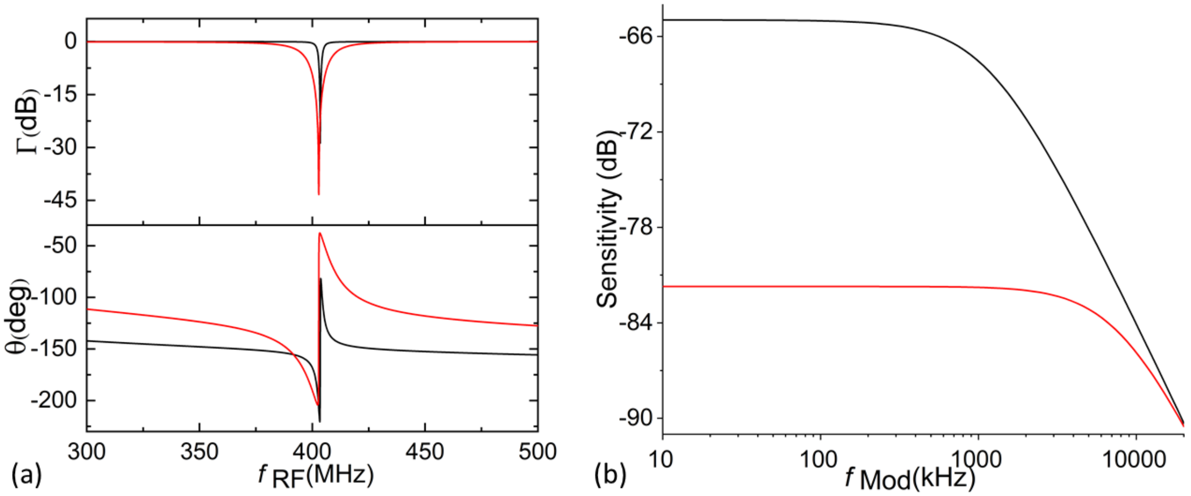

Figure S11. Simulations of matching network response and device sensitivity using two circuit models. This work - black, model used by Ibbesron[7] - red. .(a) Comparison of MN response curves tuned to matching conditions. (b) Comparison of the sensitivity to gate modulation for V_{Mod}=3.5 mV RMS.

References

1. VISHAY INTERTECHNOLOGY, I. Frequency response of thin film chip resistors https:https://www.vishay.com/docs/49427/vse-tn00.pdf (2009).
2. Frake, J. C. *et al.* Radio-frequency capacitance spectroscopy of metallic nanoparticles. *Sci. Reports* **5**, 10858 (2015).
3. Filmer, M. J., Zirkle, T. A., Chisum, J., Orlov, A. O. & Snider, G. L. Using single-electron box arrays for voltage sensing applications. *Appl. Phys. Lett.* **116**, 213103 (2020).
4. Zirkle, T. A. *et al.* Radio frequency reflectometry of single-electron box arrays for nanoscale voltage sensing applications. *Appl. Sci.* **10**, DOI: 10.3390/app10248797 (2020).
5. Coilcraft Industries, inc. Spice model -0805cs https://www.coilcraft.com/getmedia/c86a99da-e310-4932-bb03-09af08346f8a/spice_0805cs.pdf (2012).
6. Gonzalez-Zalba, M. F., Barraud, S., Ferguson, A. J. & Betz, A. C. Probing the limits of gate-based charge sensing. *Nat. Commun.* **6**, 8 (2015).
7. Ibberson, D. J. *et al.* Low-temperature tunable radio-frequency resonator for sensitive dispersive readout of nanoelectronic devices. *Appl. Phys. Lett.* **114**, 123501 (2019).
